# CLP36 promotes p53 deficient sarcoma progression through suppression of atrophin-1 interacting protein-4 (AIP-4)-dependent degradation of YAP1

**DOI:** 10.7150/thno.72365

**Published:** 2022-07-04

**Authors:** Yixuan Lu, Yongxin Mu, Ju Chen, Xinyuan Guan, Ling Guo, Chuanyue Wu

**Affiliations:** 1 Guangdong Provincial Key Laboratory of Cell Microenvironment and Disease Research, Shenzhen Key Laboratory of Cell Microenvironment, Department of Biology, and Academy for Advanced Interdisciplinary Studies, Southern University of Science and Technology, Shenzhen, 518055, China.; 2 Department of Clinical Oncology, Li Ka Shing Faculty of Medicine, The University of Hong Kong, Hong Kong, 999077, China.; 3 Department of Medicine, University of California, San Diego, La Jolla, CA 92093, USA.; 4 Department of Pathology, School of Medicine, University of Pittsburgh, Pittsburgh, PA 15261 USA.

**Keywords:** Sarcoma, CLP36, p53, YAP1, AIP-4

## Abstract

**Background:** p53 deficiency is a key causal factor for tumor development and progression. p53 acts in this process through, at least in part, cooperation with YAP1 but the underlying molecular mechanism is incompletely understood. In this paper, we show that CLP36, an actinin-binding cytoskeletal protein, links p53 deficiency to up-regulation of YAP1 expression and sarcoma progression.

**Methods:** Immunohistochemical staining and Western blotting were used to investigate the effect of p53 deficiency on CLP36 expression in sarcoma tissues and cells. Furthermore, molecular, cellular, and genetic knockout and knockdown approaches were employed to investigate the functions of CLP36 in regulation of sarcoma cell behavior in culture and tumor growth in mice. Finally, biochemical approaches were used to investigate the molecular mechanism by which CLP36 regulates the malignant behavior of p53 deficient sarcoma cells.

**Results:** We have found that the expression of CLP36 is up-regulated in response to loss of p53 in sarcoma tissues and cells. Depletion of CLP36 inhibited malignant behavior of p53 deficient sarcoma cells. Furthermore, knockout of CLP36 in mice markedly inhibited p53 deficiency-induced tumorigenesis and improved the survival of the p53 deficient mice. Mechanistically, CLP36 promoted p53 deficiency-induced tumorigenesis through inhibition of E3 ligase atrophin-1 interacting protein-4 (AIP-4)-dependent proteasomal degradation of YAP1 and consequently increase of YAP1 expression.

**Conclusions:** Our results reveal a crucial role of CLP36 in linking p53 deficiency to up-regulation of YAP1 expression and sarcoma progression. Our findings suggest that therapeutic targeting the CLP36/YAP1 signaling axis may provide an effective strategy for alleviation of p53 deficient sarcoma progression.

## Introduction

p53 is a key tumor suppressor that regulates diverse cellular processes through activating specific gene expression under various stress signals 1-3. p53 mutations that cause its aberrant expression (e.g., deficiency) or functions have been found in more than 50% of human cancers 3-6. The importance of p53 deficiency in tumorigenesis is exemplified by studies on genetic knockout (KO) of p53 from mice, which cause spontaneous tumors in multiple tissues, including sarcomas and T-cell thymic lymphoma, and death of the mice within six months 7, 8. p53 deficiency in cells alters gene expression, which, through interactions and cooperation with other signaling pathways, results in malignant behavior (e.g., increased cell proliferation, focus formation, anchorage-independent growth, etc.) and consequently tumorigenesis.

One of the critical signaling pathways that interacts and cooperates with p53 in tumorigenesis is that of Yes-associated protein 1 (YAP1), a transcriptional coactivator intimately involved in regulating cell contact inhibition, proliferation, cytoskeletal remodeling, and tissue and organ growth 9. Depending on the status of p53, YAP1 can either drive or suppress tumorigenesis 10. In particular, loss of p53 is known to activate YAP1 signaling, allowing it to drive tumorigenesis 11-14. How loss of p53 activates YAP1 signaling, however, is incompletely understood.

Previous studies have identified several signaling pathways, including both Hippo/Large tumor suppressor kinase (LATS)-dependent and -independent pathways 15, 16, that control YAP1 expression and signaling. The Hippo/LATS signaling pathway consists of a cascade of core protein kinases, including Ste20-like kinases 1/2 and LATS1/2, which can phosphorylate YAP1, leading to cytoplasmic sequestration and E3 ubiquitin ligase (e.g., β-Transducin repeat-containing proteins (β-TRCP))-mediated proteasomal degradation of YAP1 9, 17. Additionally, substantial evidence suggests that YAP1 expression and signaling can also be regulated by Hippo/LATS-independent pathways, which are often mediated by alterations of F-actin 18-23. Previous studies by us and others have shown that atrophin-1 interacting protein-4 (AIP-4), an E3 ubiquitin ligase, can act downstream of F-actin to catalyze ubiquitination and proteasomal degradation of YAP1 24-26. The relative contributions of Hippo/LATS-dependent and -independent pathways to the regulation of YAP1 appear to be dependent on cell type and upstream stimulus.

CLP36 (also known as PDZ and LIM domain protein 1) is a ubiquitously expressed α-actinin-binding cytoskeletal protein 27-30. Structurally, CLP36 contains an N-terminal PDZ domain, a C-terminal LIM domain, and a ZASP-like motif (ZM) between the PDZ and LIM domains. Previous studies by us and others have shown that CLP36, through its PDZ domain and to a less extent the middle ZASP-like motif (ZM) 31, interacts with α-actinin 30-32, an actin cross-linking protein 33. Through interaction with α-actinin, CLP36 localizes to F-actin 30, where it cooperates with other cytoskeletal proteins to regulate actin cytoskeletal organization 34-37**.** The importance of CLP36 in actin cytoskeletal organization has been illustrated by Tamura and co-workers, who showed that knockdown of CLP36 from BeWo cells resulted in loss of actin stress fibers and focal adhesions 29. Previous studies using various cancer cell lines suggest that alteration of CLP36 expression can impact the behavior of cancer cells in a context-dependent manner 38. To better understand the function of CLP36 in tumorigenesis *in vivo*, we have genetically knocked out CLP36 in mice and investigated its role in p53 deficiency-induced tumorigenesis. Our results demonstrate a crucial role of CLP36 in promoting p53 deficiency-induced tumorigenesis and shed light on the molecular mechanism through which CLP36 functions in this process.

## Methods

### Mice

CLP36^-/-^ mice were generated as follows. Genomic DNA fragments of *CLP36* were amplified from R1 embryonic stem (ES) cells using polymerase chain reaction (PCR) and used to construct the CLP36-targeting construct as previously described 39. Briefly, one *loxP* site was inserted into intron 2, and a second *loxP* site, together with a neomycin cassette (*neo*) flanked by *frt* sites, was inserted into intron 3. The targeting construct was verified by sequencing and linearized with the restriction enzyme *Not*I and electroporated into R1 ES cells derived from 129/SvJ mice (UCSD Transgenic and Gene Targeting Core, La Jolla, CA, USA).

Targeted ES cells were identified by Sothern blotting analysis as previously described 39. Briefly, genomic DNA from G418-resistant ES cell clones was digested with restriction enzyme *Hin*dIII and hybridized with the radiolabeled probe generated by PCR using mouse genomic DNAs and specific *CLP36* primers (forward primer, 5'-TGTGTGAGCAATGTGTTGTGA-3'; reverse primer, 5'-AGGTTGGGGCTGTGGATAC-3'). The wild type allele is represented as an 8.4 kb band, whereas a 3.8 kb band represents the targeted allele. ES cells from a homologous recombinant clone were then microinjected into C57BL/6 blastocysts. Male chimeras were bred with female Black Swiss mice (Taconic Inc., Hudson, NY, USA) to generate germline-transmitted heterozygous mice with a *neo* cassette, which were further confirmed by Southern blot analysis of mouse tail DNA.

The germline-transmitted heterozygous mice with a neo cassette were crossed with FLPase deleter mice 40 to delete the neo cassette to generate the floxed heterozygous CLP36 mice. The global heterozygous CLP36 KO mice were generated by crossing floxed heterozygous CLP36 mice with the protamine-Cre transgenic mouse line 41. Offspring were genotyped by PCR analysis of mouse tail DNA with the genotyping CLP36 forward primers P1: 5'-TCCCATGGACCAAGCATATT-3'; reverse P2: 5'-CCAGGAGAACCAATGAGGAA-3'; and P4: 5'-GTAGGGCACTGAAGGGAACA-3'.

The p53^+/-^ mice (B6;129S4-*Trp53^tm5Tyj^*/J Stock No: 008361) were obtained from Jackson Laboratory. The double transgenic CLP36^-/-^ p53^-/-^ mice were generated by mating the two individual strains. Genotyping of the alleles was performed by PCR using the following oligonucleotide primers: *Trp53* wild forward primer 5'-AGCCTGCCTAGCTTCCTCA-3'; *Trp53* common reverse primer 5'-TCTTGGAGACATAGCCACACTG-3'; *Trp53* mutant forward primer 5'- GCCATACCACATTTGTAGAGGTT-3'; *CLP36* forward primer 5'- TCCCATGGACCAAGCATATT-3'; *CLP36* reverse primer P2 5'- CCAGGAGAACCAATGAGGAA-3'; *CLP36* reverse primer P4 5'- GTAGGGCACTGAAGGGAACA-3'. The recombinant alleles were analyzed using genomic DNA extracted from the tips of mouse tails. The mouse work was performed with the approval of the Institutional Animal Care and Use Committee, Southern University of Science and Technology.

### Cell culture

Human Saos‐2 (TCHu114) osteosarcoma cells were purchased from the Typical Culture Preservation Committee of the Chinese Academy of Sciences, Shanghai, China. The cells were cultured in McCoy's 5A medium (HyClone Laboratories) supplemented with 10% fetal bovine serum (FBS, PAN BIOTECH) and 1% penicillin-streptomycin (P/S, HyClone Laboratories). Human HT1080 fibrosarcoma cells were provided by Dr. Yi Deng (Southern University of Science and Technology), and cultured in minimum essential medium (MEM, CORNING) supplemented with 10% FBS, 1× non-essential amino acid solution (NEAA, Gibco) and 1% P/S. Human U2OS osteosarcoma cells were provided by Dr. Bin Tang (Southern University of Science and Technology), and cultured in High-Glucose Dulbecco's modified Eagle's medium (DMEM, CORNING) supplemented with 10% FBS and 1% P/S. The cells were cultured at 37 °C with 5% CO_2_ and harvested with 0.25% trypsin-EDTA (Gibco) before reaching full confluence.

### Protein extraction and Western blotting

Mouse tissues and cells (as specified in each experiment) were isolated and homogenized in pre-cold 1× LDS sample buffer (M00676, GenScript) containing the protease and phosphatase inhibitor cocktails (Roche Diagnostics GmbH, Mannheim, Germany). Protein extracts were separated by sodium dodecyl sulfate polyacrylamide gel electrophoresis and analyzed by Western blotting. Primary antibodies were used as follows: CLP36 (1:1000; Rabbit monoclonal, Abcam, ab129015), YAP1 (1:1000, Mouse monoclonal, Santa Cruz, sc-101199), AIP-4 (1:1000, Rabbit polyclonal, Cell Signaling, 12117S), Phospho-YAP (Ser127) (1:1000, Rabbit polyclonal, Cell Signaling, 4911S), LATS1 (1:1000, Rabbit polyclonal, Cell Signaling, 3477S), Phospho-LATS1 (Thr1079) (1:1000, Rabbit polyclonal, Cell Signaling, 8654S), TAZ (1:1000, Rabbit polyclonal, Cell Signaling, 4883S), p53 (1:1000, Rabbit polyclonal, Cell Signaling, 2527S) and GAPDH (1:5000, Mouse monoclonal, Santa Cruz, sc-47724HRP). The protein levels of GAPDH were used as a loading control. The intensities of the protein bands were quantified by densitometry and analyzed with Image J from three independent experiments.

### RNA extraction and Reverse Transcription-Quantitative Polymerase Chain Reaction (RT-qPCR)

Total RNA was extracted from cells with TRIzol (Invitrogen). First-strand cDNA was prepared by reverse transcription with Superscript II reverse transcriptase (Invitrogen) and oligo (dT) primers and stored at -20 °C. RT-qPCR was performed using SYBR ® Premix Ex TaqTM II with an ABI 7500 QPCR System. The mRNA levels of GAPDH were used as an internal control, quantified in parallel with those of the target genes. Normalization and fold changes were calculated using the ΔΔCt method. Primers used were as follows: human GAPDH forward primer 5'-CCAGAACATCATCCCTGCCTCTACT-3'; human GAPDH reverse primer 5'-GGTTTTTCTAGACGGCAGGTCAGGT-3'; human CLP36 forward primer 5'-GCTGGCCTCTACTCTTCTGAA-3'; human CLP36 reverse primer 5'-GCTGAGCATGGTCTAAGGGT-3'; human YAP1 forward primer 5'-TAGCCCTGCGTAGCCAGTTA-3'; human YAP1 reverse primer 5'-TCATGCTTAGTCCACTGTCTGT-3'; mouse GAPDH forward primer 5'-TACAGCAACAGGGTGGTGGAC-3'; mouse GAPDH reverse primer 5'-TGGGATAGGGCCTCTCTTGCT-3'; mouse CLP36 forward primer 5'-TCGATGGGGAAGATACCAGCA-3'; mouse CLP36 reverse primer 5'-TCTGTTCAGACCTGGATACTGTG-3'; mouse YAP1 forward primer 5'-GTCCCACTCGCGACAGGCCA-3'; mouse YAP1 reverse primer 5'-CGGCAGGGCCAGAGACAACG-3'.

### Tumor analysis and immunohistochemical staining

Human osteosarcoma tissue microarray (TMA, LBO804e) comprising 40 cases in duplicate was purchased from Tbsbio (Xi'an, China). The clinical information (age, sex, type, etc.) of the patient and specimens was provided by Tbsbio (Xi'an, China) and is included in Supplementary Table 1.

Mice that died or were sacrificed were subjected to complete necropsy. Solid tumor samples were surgically removed, fixed in 10% formalin, and embedded in paraffin. The samples were cut to 5 μm thick, analyzed by hematoxylin and eosin (H&E) staining, and examined under a microscope. Thymic lymphoma tissues were collected from the thoracic cavity of the mice, and most of the sarcoma tissue encompassed the leg of mice. Other solid tumors that were collected included head and neck, spine, lung tumors, and sarcoma in the back.

For tissue staining, tissue sections were rehydrated. Pretreatment for antigen retrieval was performed in boiled Citric Acid buffer (MXB Biotechnologies) for 2 min. Endogenous tissue peroxidase was blocked by treating the sections with 0.3% H_2_O_2_. The human TMA sections were stained with antibodies against CLP36 (1:100, Rabbit polyclonal, Sigma-Aldrich, HPA017010), YAP1 (1:200, Mouse monoclonal, Santa Cruz, sc-101199) or p53 (1:100, Rabbit monoclonal, Cell Signaling, 2527S), and images were captured and quantified for every single sample. We divided the tumor points into p53 positive (p53+) (n = 5) and p53 negative (p53-) (n = 31) groups based on the expression of p53. Mouse tissue sections were stained with primary antibodies against CLP36 (1:100; Mouse monoclonal, Santa Cruz, sc-393084) or YAP1 (1: 200, Mouse monoclonal, Santa Cruz, sc-101199), secondary HRP-polymer anti-mouse or anti-rabbit secondary antibodies (MXB Biotechnologies, MaxVisionTM), 3,3'-Diaminobenzidine (DAB) and then counterstained with hematoxylin.

The images of tissue staining were captured with a digital camera (DS-Fi1c; Nikon) and NIS-Elements F Ver4.30.01 image analysis software (Nikon) with a × 40 or × 20 objective. The blue-counterstained nuclei were excluded, and the integrated density of three random fields per slide under a × 40 objective was analyzed by Image J.

### Lentiviral vector generation and infection

The pLKO.1 (Addgene, 10878), psPAX2 (Addgene, 12260) and pMD2.G (Addgene, 12259) vectors were from Addgene. The pLKO.1 vector expressing short hairpin RNA (Sh-RNA) targeting human p53, human CLP36, mouse CLP36 or scrambled shRNA (Sh-NC) sequence were generated using the following sequences: ShNC 5'-ACGCATGCATGCTTGCTTT-3'; Sh-p53 5'-GTCCAGATGAAGCTCCCAGAA-3'; h-Sh-CLP36 5'-GCCTTGGTTAATTGACTCACA-3'; m-Sh-CLP36 5'-ACAAATGTGGAACTGGTATTG-3'; m-Sh-NC 5'-ACGCATGCATGCTTGCTTT-3'.

To generate protein expression vectors encoding human p53, CLP36, YAP1, and CLP36-ABD mutant (amino acid 69-137,164-329), complementary DNAs encoding the corresponding protein sequences were cloned into the pLVX-3xflag vectors. To produce lentiviruses, pLKO.1 encoding the shRNAs or lentiviral expression vectors encoding human p53, CLP36, YAP1, and CLP36-ABD mutant sequences were co-transfected with psPAX2 and pMD2.G into 293T cells using Lipofectamine 3000 transfection kit (Invitrogen). After 48 h, the media containing lentiviral particles were harvested. For lentiviral infection, cells in 70% confluence were cultured with fresh medium containing lentivirus (as specified in each experiment) in the presence of 8 μg/mL polybrene.

### Generation of CLP36 KO sarcoma cells

CLP36 KO sarcoma cells were generated with CRISPR/Cas9-mediated gene-editing system. Two guide RNA oligos designed to target the sequence of 5'- AGTCCTTGCCGCCCACGAGG-3' and 5'-AGCAGCCTCTCGCCATTTCC-3' located in the exon 1 of human *CLP36* gene were cloned into pSpCas9n (BB)- 2A-GFP (PX461 containing cas9n was obtained from Dr. Feng Zhang, Addgene #48140) via *Bbs*I sites. The plasmids targeting the *CLP36* gene were transfected into Saos-2 and HT1080 sarcoma cells using Lipofectamine 3000 transfection kit (Invitrogen). Singular green fluorescent protein (GFP)-positive cells were sorted into 96-well plates with FACS sorter (BD FACS AriaTMIII). Individual CLP36 KO colonies were analyzed and confirmed by Western blotting. The CLP36 KO Saos-2 or HT1080 cells were cultured as described above. For inhibition with MG132 or Leupeptin, cells were treated with MG-132 (Selleck, 10 μM) or Leupeptin (Selleck, 10 μM) for 8 h and then collected and analyzed as specified in each experiment.

### RNA interference

siRNAs against human AIP-4 or β-TRCP were synthesized by IGEbio (Guangzhou, China). The sequences of siRNA were as follows: human AIP-4 5'- GCCTATGTTCGGGACTTCAAA-3', β-TRCP 5'- AAGUGGAAUUUGUGGAACAUC-3', YAP1 5'- CCCAGTTAAATGTTCACCAAT-3', TAZ 5'-AGAGGTACTTCCTCAATCA-3', and control 5'-ACGCATGCATGCTTGCTTT-3'. Cells in each well of a six-well culture dish were transfected with 25 pM siRNA and 5 μL Lipofectamine RNAiMAX Transfection Reagent (Life Technologies).

### Co-immunoprecipitation

Cells (as indicated in each experiment) were harvested and homogenized in the lysis buffer (Beyotime, P0013) supplemented with protease inhibitor cocktail (Roche, 04693132001) at 4 ºC for 30 min. Protein concentration was measured using a Pierce BCA Protein Assay kit (Thermo Fish Scientific, 23227). Cell lysates were pre-cleared with 20 μL protein A/G-Sepharose beads (Santa Cruz, sc-2003). The pre-cleared cell lysates were incubated with 2 μg YAP1 (Mouse monoclonal, Santa Cruz, sc-101199), CLP36 (Mouse monoclonal, Santa cruz, sc-393084) or normal mouse IgG (Santa Cruz, sc-2025) antibodies and 30 μL of protein A/G-Sepharose beads at 4 °C overnight, followed by washing three times with the PBS and one time with the lysis buffer. All samples were boiled with 2× LDS sample buffer (M00676, GenScript) and then analyzed by Western blotting.

### Immunofluorescence

Cells (as specified in each experiment) were seeded on coverslips in 24-well plates (2 × 10^4^ cells per well). After culturing overnight, the cells were fixed with 4% paraformaldehyde (PFA), washed three times with PBS, immersed in 0.1% Triton X-100 in PBS for 10 min at room temperature, and then washed three times with PBS again and incubated with YAP1 (1:1000, Mouse monoclonal, Santa Cruz, sc-101199) antibodies at 4 °C overnight. Cells were then washed three times with PBS and incubated with Alexa Flours-594 conjugated anti-mouse IgG secondary antibodies (1:500, Invitrogen, A-11032) at room temperature. Cells were co-stained with DAPI (4,6-diamidino-2-phenylindole) for 30 min at room temperature. Images were acquired at 21 °C using an SP8 confocal fluorescence microscope (× 20 dry objective 0.7 numerical aperture (NA) or × 40 dry objective 0.85 NA; Leica) with Leica X Version: 1.1.0.12420 image software. The percentages of cells with positive nuclear YAP1 staining were calculated by analyzing at least 50 cells from each cell type.

### Generation of mouse p53 null sarcoma cells and allograft assay

For generation of mouse p53 null sarcoma cells, sarcoma tissues from p53^-/-^ mice were minced and enzymatically dissociated with 0.25% trypsin. The cells were then washed with PBS and cultured. Adherent sarcoma cells were maintained in High-Glucose Dulbecco's Modified Eagle Medium (DMEM, CORNING) supplemented with 10% FBS and 1% penicillin-streptomycin.

For allograft experiments, p53 null sarcoma cells from the mouse were dissociated, counted, and suspended in cold PBS. The cells (as specified in each experiment) were injected subcutaneously into rear flank of the C57BL/6 mice (5-weeks old; 1 × 10^6^ cells/point). After 14 days, the mice were imaged after administration of anesthesia, and the tumors were individually collected, weighed and analyzed.

### CCK-8 cell proliferation assay

The cells (as specified in each experiment) were plated in 96-well culture dishes with a density of 2000 cells per well. Cells were cultured for three days unless otherwise specified, then treated with 10 μL Cell Counting Kit-8 (CCK-8) solution (Beyotime, Shanghai, China) in 100 μL medium, and incubated for 1 h at 37 °C with 5% CO_2_. The absorbance values at 450 nm were quantified with a microplate reader (EPOCH2). Average absorbance values from three wells were calculated. For each cell group, data from three independent experiments were analyzed.

### Focus formation assay

The cells (as specified in each experiment) were plated in six-well tissue culture dishes with a density of 1000 cells per well in triplicate. After culturing for 3 weeks, foci were stained with crystal violet and foci over 50 cells were counted under microscope. The percentage of focus formation was calculated by dividing the number of the foci with the number of seeded cells.

### Soft agar assay

The cells (as specified in each experiment) mixed with complete medium containing 0.3% agar were plated over a base layer of solidified complete medium containing 0.8% agar in six-well tissue culture dishes in triplicate (50000 cells/well). The cells were incubated at 37 °C with 5% CO_2_. The cell culture medium was replenished every 3-4 days. After 3-4 weeks of culture, the cells were stained with 200 μL of nitroblue tetrazolium chloride solution per well and incubated overnight at 37 °C with 5% CO_2_. Colonies over 50 µm (1.11 µm/pixel) were counted under a microscope (Olympus mvx10, × 6.3 objective). The percentage of colony formation was calculated by dividing the number of the colonies with the number of seeded cells.

### Cell migration assay

The cells (as specified in each experiment) were washed with PBS, trypsinized, and then mixed with the complete medium. The cells were pelleted by centrifugation and washed once with the medium without FBS. The cells were seeded into the transwell motility chambers with 300 μL medium without FBS (40000 cells/chamber; the pore size of the membrane inserts = 8 μm, Costar 3422), while 700 μL complete medium was added into each well of the 24-well plate in which the transwell motility chambers were placed. After incubation at 37 °C with 5% CO_2_ for 24 h, the cells on the upper side of the membrane inserts were removed, and the cells that had migrated to the lower side were fixed with 4% paraformaldehyde and stained with DAPI (4,6-diamidino-2-phenylindole) for 30 min at room temperature. Migrated cells in five random fields were counted with Image J, and data from three independent experiments were analyzed. The images were captured with a digital camera (DS-U3, Nikon) and NIS-Elements F Ver4.30.01 image analysis software (Nikon) with a × 20 objective.

### Statistical analysis

Statistical significance was analyzed with Prism 8 software. The results of three independent experiments were analyzed, and the standard deviation (S.D.) was calculated unless otherwise specified. The two-tailed unpaired Student's *t*-test was used to compare two groups of data, and one-way ANOVA was used to compare more than two groups, with a *p*-value < 0.05 considered statistically significant. Statistically significant changes were indicated with asterisks (* *p* < 0.05; ** *p* < 0.01; *** *p* < 0.001; **** *p* < 0.0001.).

Survival functions were plotted using the Kaplan-Meier method, and a comparison of survival functions was performed by the log-rank test. A *p*-value < 0.05 was considered statistically significant.

## Results

### p53 deficiency up-regulates CLP36 expression *in vitro* and *in vivo*

**Figure 1:**
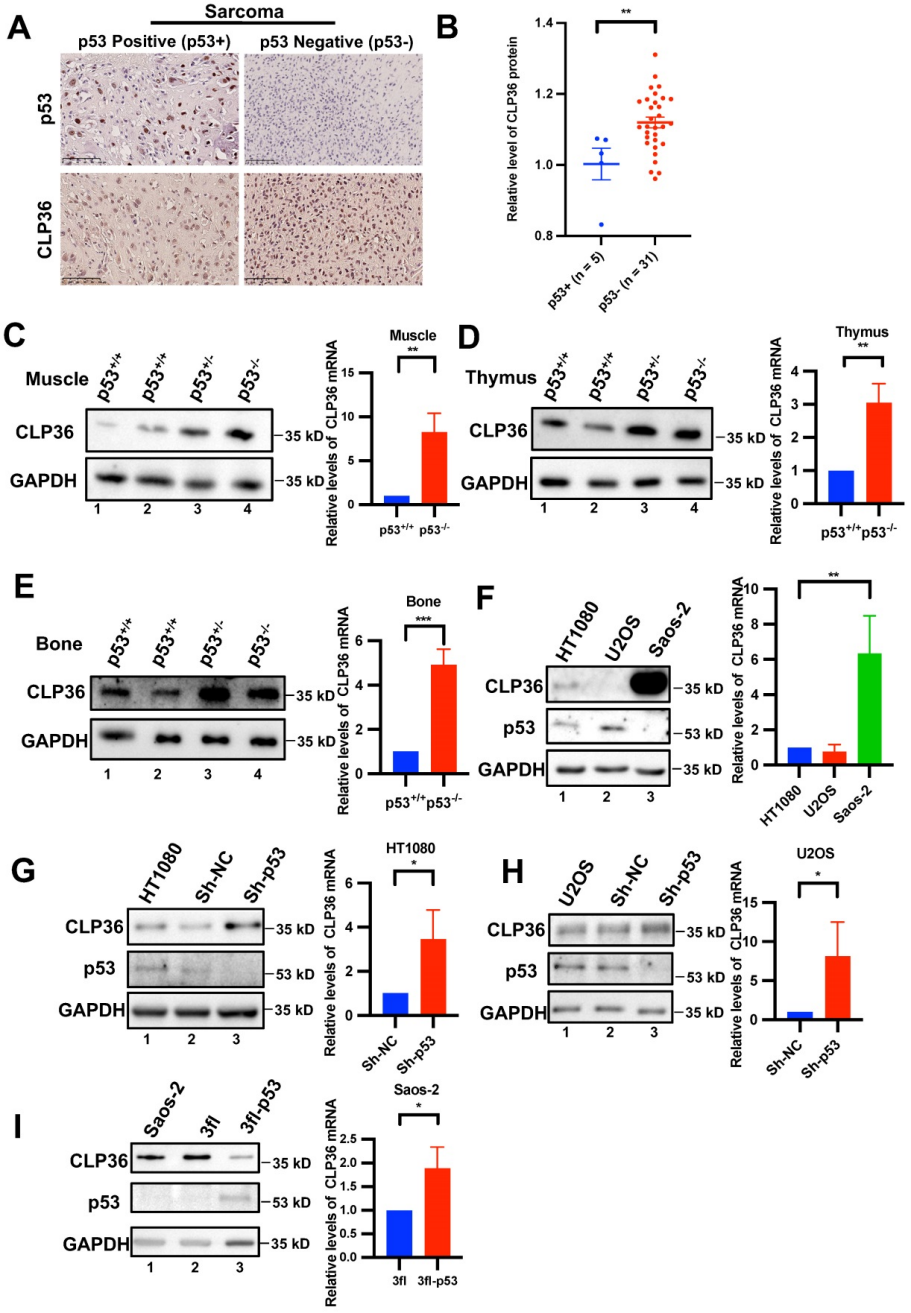
** p53 negatively regulates CLP36 expression. A**, **B** human osteosarcoma tissues from a tissue microarray (Tbsbio, Xi'an, China) were stained with antibodies for p53 or CLP36 as described in the “Methods” (Scale bar = 100 µm) (**A**). The clinical information of the tissue samples was shown in Supplementary Table 1. The mean intensity of CLP36 in the p53 positive group (n = 5) was compared to that in the p53 negative group (n = 31) (**B**). **C**, **D**, **E** Skeletal muscle, thymus and bone normal tissue protein and RNA were extracted from p53 wild type (p53^+/+^) or p53 deficient (p53^-/-^) mice. The protein levels of CLP36 were analyzed by Western blotting. Right panel, the mRNA levels of CLP36 were analyzed by RT-PCR and compared to those in the p53^+/+^ tissues (normalized to 1; n = 3). **F** HT1080, U2OS and Saos-2 cells were analyzed by Western blotting for CLP36, p53 or GAPDH. Right panel, the mRNA levels of CLP36 in the U2OS and Saos-2 cells were analyzed by RT-PCR and compared to that in the HT1080 cells (normalized to 1; n = 3). **G, H** HT1080 (**G**) or U2OS (**H**) cells were infected with control (Sh-NC) or p53 (Sh-p53) shRNA lentivirus for five days. The cells were analyzed by Western blotting as indicated. Right panels, the mRNA levels of CLP36 in the Sh-p53 cells were analyzed by RT-PCR and compared to those in the Sh-NC cells (normalized to 1; n = 3). **I** Saos-2 cells were infected with 3xflag-tagged p53 (3fl-p53) or 3xflag vector(3fl) lentivirus for three days. The cells were analyzed by Western blotting as indicated. Right panel, the mRNA level of CLP36 in the 3fl-p53 cells was analyzed by RT-PCR and compared to that in the 3fl cells (normalized to 1; n = 3). Data in **B** are shown as mean ± SEM. Data in **C**, **D**, **E**, **F**, **G**, **H**, and **I** are presented as mean ± S.D. Statistical significance was calculated using a two-tailed unpaired Student's *t*-test, *p < 0.05; **p < 0.01.

During our initial studies, we found that CLP36 expression was frequently increased in p53 negative human sarcoma tissues compared with that in p53 positive sarcoma tissues (Figures 1A-B). These results raised an interesting possibility that p53 deficiency may promote CLP36 expression. To test this possibility, we analyzed CLP36 expression in muscle tissues of p53 KO mice by Western blotting. The results showed that p53 deficiency did significantly increase the mRNA and protein levels of CLP36 in mouse muscle (Figure 1C, compare lanes 3 and 4 with lanes 1 and 2). Similar results have been obtained with the thymus and bone tissues of p53 KO mice (Figures 1D-E, compare lanes 3 and 4 with lanes 1 and 2). These results suggest that p53 deficiency promotes CLP36 expression. To further investigate this, we analyzed the effects of altering the expression of p53 on CLP36 expression in p53-expressing HT1080 fibrosarcoma cells and U2OS osteosarcoma cells as well as p53 null Saos-2 osteosarcoma cells. The p53-expressing sarcoma cells (i.e., HT1080 and U2OS) express relatively modest or low levels of CLP36 (Figure 1F, lanes 1 and 2), whereas the p53 null sarcoma cells (i.e., Saos-2) express a significantly higher level of CLP36 (Figure 1F, lane 3). Importantly, knockdown of p53 from the p53-expressing sarcoma cells (i.e., HT1080 and U2OS), like KO of p53 in mice (Figures 1C-E), significantly increased the mRNA and protein levels of CLP36 (Figures 1G-H). These results suggest that p53 deficiency promotes CLP36 expression through, at least in part, up-regulation of *CLP36* gene transcription in these cells. Consistent with a negative regulatory role of p53 on CLP36 expression, overexpression of p53 in the p53 null sarcoma cells (i.e., Saos-2) significantly reduced the protein level of CLP36 (Figure 1I), although in this case, the mRNA level of CLP36 was increased in response to overexpression of p53 (Figure 1I), indicating that in these cells p53 overexpression-induced down-regulation of CLP36 expression is at the post-transcriptional rather than the transcriptional level. Collectively, these results suggest that p53 is a negative regulator of CLP36 expression, and depending on the cell context, p53 can negatively regulate CLP36 expression through multiple mechanisms (e.g., at transcriptional and/or post-transcriptional levels).

### Loss of CLP36 inhibits p53 deficiency-induced tumorigenesis

**Figure 2:**
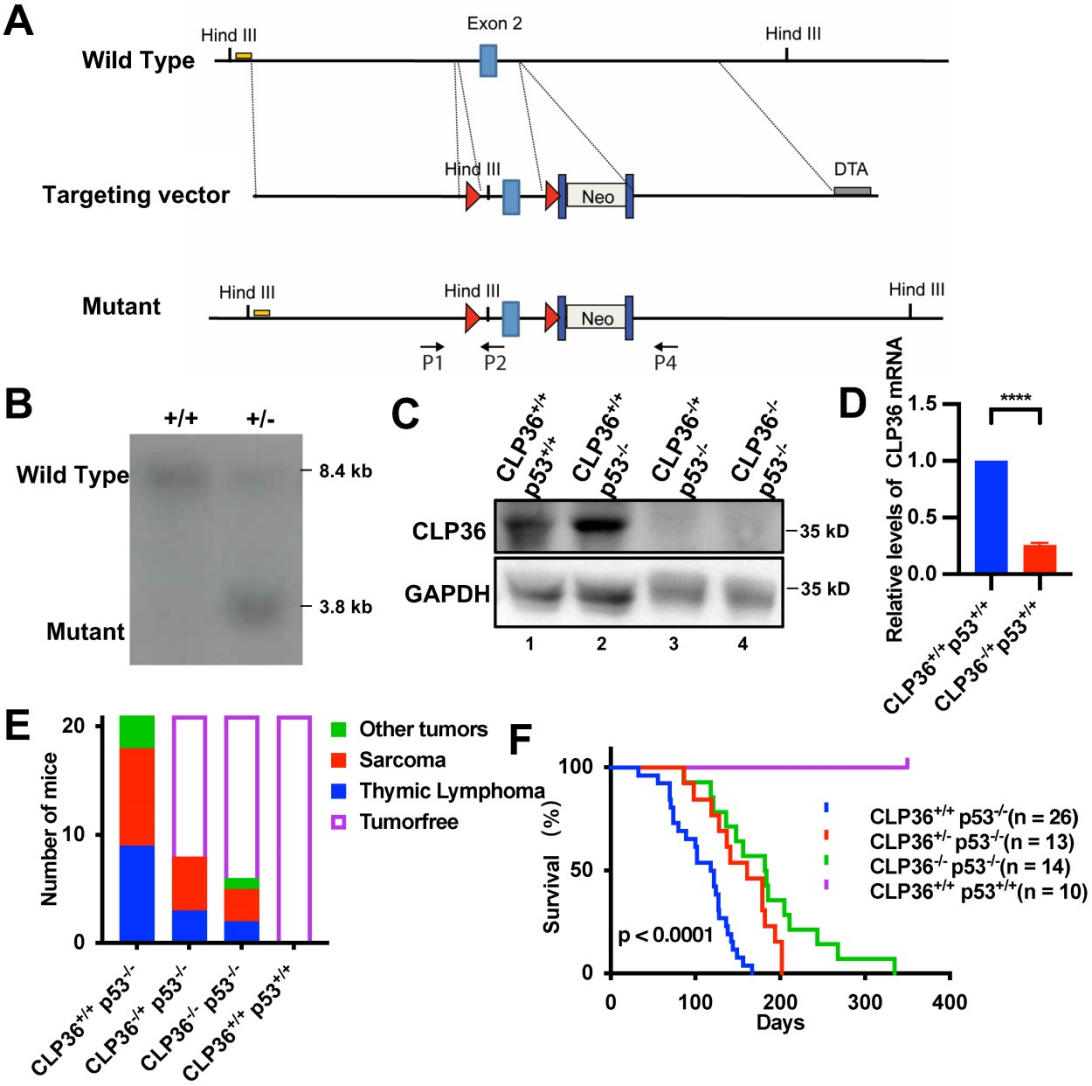
** KO of CLP36 inhibits p53 deficiency-induced tumor growth in mouse. A** The conditional KO allele of the *CLP36* gene was generated as described in the “Methods”. A restriction map of the relevant genomic region of *CLP36* (top), the targeting vector (middle), and the mutant locus after homologous recombination (bottom) are shown. The light blue rectangles indicate the targeted exon 2. Red triangles indicate *LoxP* sites. Dark blue rectangles indicate *FRT* sites. Yellow line indicates the southern blot probe region. *DTA*, Diphtheria Toxic A chain gene; *Neo*, Neomycin resistance gene. Arrows indicate the position for genotyping primers. **B** DNA from electroporated embryonic stem (ES) cells were digested with *Hin*dIII and analyzed by Southern blotting as described in the “Methods”. The 8.4 kb and 3.8 kb bands represent wild type (WT) and mutated (MT) alleles. **C** Skeletal muscle tissue protein samples were extracted from the mice (as indicated) and analyzed by Western blotting with antibodies for CLP36 and GAPDH. **D** The mRNA level of CLP36 in the CLP36^-/+^ p53^+/+^ muscle tissues was analyzed by RT-PCR and compared to that in the CLP36^+/+^ p53^+/+^ muscle tissues (normalized to 1; n = 3). **E** The numbers of the CLP36^+/+^ p53^-/-^, CLP36^+/-^ p53^-/-^, CLP36^-/-^ p53^-/-^ and CLP36^+/+^ p53^+/+^ mice with tumors (as indicated) were counted, and the tumors were dissected at the age of six months (n = 21 mice for each group). Red, sarcoma; blue, thymic lymphoma; green, other tumors including head and neck tumor, spine tumor or lung tumor; blank, tumor-free. **F** Survival of the CLP36^+/+^ p53^-/-^ (n = 26), CLP36^+/-^ p53^-/-^ (n = 13), CLP36^-/-^ p53^-/-^ (n = 14), and CLP36^+/+^ p53^+/+^ (n = 10) mice was analyzed by Kaplan-Meier analysis for up to 335 days. Data in **F** were determined by log-rank test, *p* < 0.0001.

We next investigated the functional significance of p53 deficiency-induced increase of CLP36 expression. To do this, we knocked out CLP36 from p53 deficient mice and determined the effect on p53 deficiency-induced tumorigenesis. Global heterozygous CLP36 KO C57BL/6 mice (CLP36^+/-^) were generated as described in the Methods (Figures 2A-B), which were crossed to generate homozygous CLP36 KO mice (CLP36^-/-^). CLP36^-/-^ mice were fertile and appeared to develop and grow normally, indicating that CLP36 is dispensable for embryonic and postnatal development. Next, we crossed the CLP36^-/-^ mice with p53^-/-^ mice to obtain CLP36^+/-^ p53^-/-^ and CLP36^-/-^ p53^-/-^ mice. Western blotting analysis of the CLP36 level in the muscle tissues of the CLP36^+/+^ p53^-/-^ and CLP36^+/+^ p53^+/+^ mice showed that, as expected, the level of CLP36 was increased in response to loss of p53 (Figure 2C, compare lane 2 with lane 1). The CLP36 expression in the muscle tissues was dramatically reduced in the CLP36^+/-^ p53^-/-^ mice and eliminated in the CLP36^-/-^ p53^-/-^ mice compared with that in the CLP36^+/+^ p53^-/-^ mice (Figures 2C-D, compare lanes 3 and 4 with lane 2). All CLP36^+/+^ p53^-/-^ mice developed tumors by six months. Many CLP36^+/+^ p53^-/-^ mice developed spontaneous sarcomas (42.9%) or lymphomas (42.9%) (Figure 2E). Head and neck, spine, or lung tumors were detected in the remaining (14.3%) of the CLP36^+/+^ p53^-/-^ mice (Figure 2E). By marked contrast, the majorities of the CLP36^+/-^ p53^-/-^ (61.9%) and CLP36^-/-^ p53^-/-^ (71.43%) mice were tumor-free at the same age (Figure 2E). Concomitant to the reduction of tumorigenesis, the survival rate of the CLP36^+/-^ p53^-/-^ mice and, to a greater extent, that of the CLP36^-/-^ p53^-/-^ mice were increased compared with that of the CLP36^+/+^ p53^-/-^ mice (Figure 2F). These results suggest that increased CLP36 expression is critically involved in the p53 deficiency-induced tumorigenesis and mortality of the mice.

### Depletion of CLP36 suppresses the malignant behavior of the p53 deficient sarcoma cells

To investigate the effects of depletion of CLP36 on the cellular behavior of p53 deficient sarcoma cells, we infected p53 deficient Saos-2 cells with lentiviral vectors encoding CLP36 shRNA (Sh-CLP36) or control shRNA (Sh-NC). Western blotting analysis confirmed that the level of CLP36 in the Sh-CLP36 cells was significantly reduced compared with those in the control Sh-NC or wild type Saos-2 cells (Figure S1A, compare lane 3 with lanes 1 and 2). In 2-D cell culture, the CLP36 knockdown cells proliferated at a slower rate than those of the control Sh-NC or wild type Saos-2 cells (Figure S1B). Furthermore, the ability of the CLP36 knockdown cells to form focus in culture was significantly decreased compared with those of the control Sh-NC or wild type Saos-2 cells (Figure S1C). In 3-D soft agar assay, knockdown of CLP36 dramatically inhibited the anchorage-independent growth of the p53 deficient sarcoma cells (Figure S1D). Finally, knockdown of CLP36 also reduced the migration of the p53 deficient sarcoma cells (Figure S1E).

**Figure 3:**
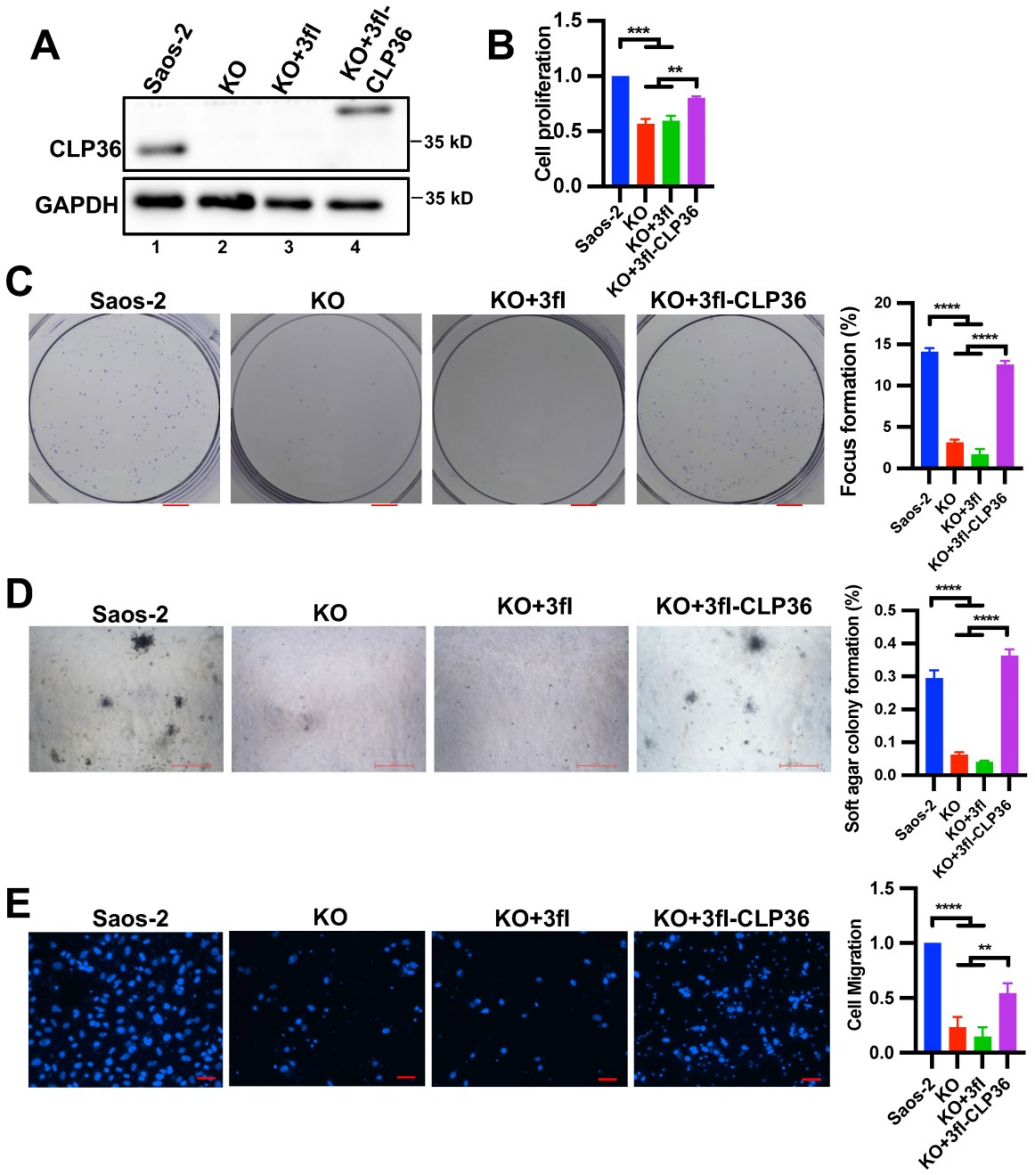
** CLP36 regulates p53 null sarcoma cell proliferation and migration.** CLP36 KO Saos-2 cells were infected with lentiviral vectors encoding 3xflag-tagged CLP36 (3fl-CLP36) or 3xflag vector (3fl) for three days. **A** The cells (as indicated) were analyzed by Western blotting with antibodies for CLP36 and GAPDH. **B** Cell proliferation was analyzed by CCK-8 assay as described in the “Methods”. The mean absorbance of the cells (as indicated) was compared to that of the Saos-2 cells (normalized to 1; n = 3). **C** Focus formation assay was performed as described in the “Methods” (scale bar = 5 mm). The mean percentage of focus formation of the cells (as indicated) was compared to that of the Saos-2 cells (right panel). **D** Anchorage-independent growth was analyzed by soft agar assay as described in the “Methods” (scale bar = 500 pixels). The mean percentage of colony formation of the cells (as indicated) was compared to that of the Saos-2 cells (right panel). **E** Cell migration was analyzed using transwell motility chambers as described in the “Methods” (scale bar = 200 pixels). Right panel, the mean number of the cells (as indicated) migrated through the membrane was compared to that of the Saos-2 cells (normalized to 1; n = 3). Data in **B**, **C**, **D**, and **E** are presented as mean ± S.D. Statistical significance was calculated using one-way ANOVA with Tukey-Kramer post-hoc analysis, **p < 0.01; ***p < 0.001; ****p < 0.0001.

To confirm that CLP36 is critical for regulating p53 deficient sarcoma cell behavior, we knocked out CLP36 from the Saos-2 cells using the CRISPR/ Cas9 gene-editing system (Figure 3A, compare lane 2 with lane 1). As expected, the CLP36 KO cells exhibited diminished abilities of cell proliferation (Figure 3B), focus formation (Figure 3C), anchorage-independent growth (Figure 3D), and migration (Figure 3E). Expression of 3xflag-tagged CLP36, but not 3xflag only, in CLP36 KO cells restored to a large extent the rate of cell proliferation (Figure 3B), focus formation (Figure 3C), anchorage-independent growth (Figure 3D), and migration (Figure 3E). Thus, consistent with a critical role of CLP36 in p53 deficiency-induced tumorigenesis *in vivo* (Figure 2), CLP36 promotes p53 deficient Saos-2 sarcoma cell proliferation, focus formation, anchorage-independent growth and migration.

**Figure 4:**
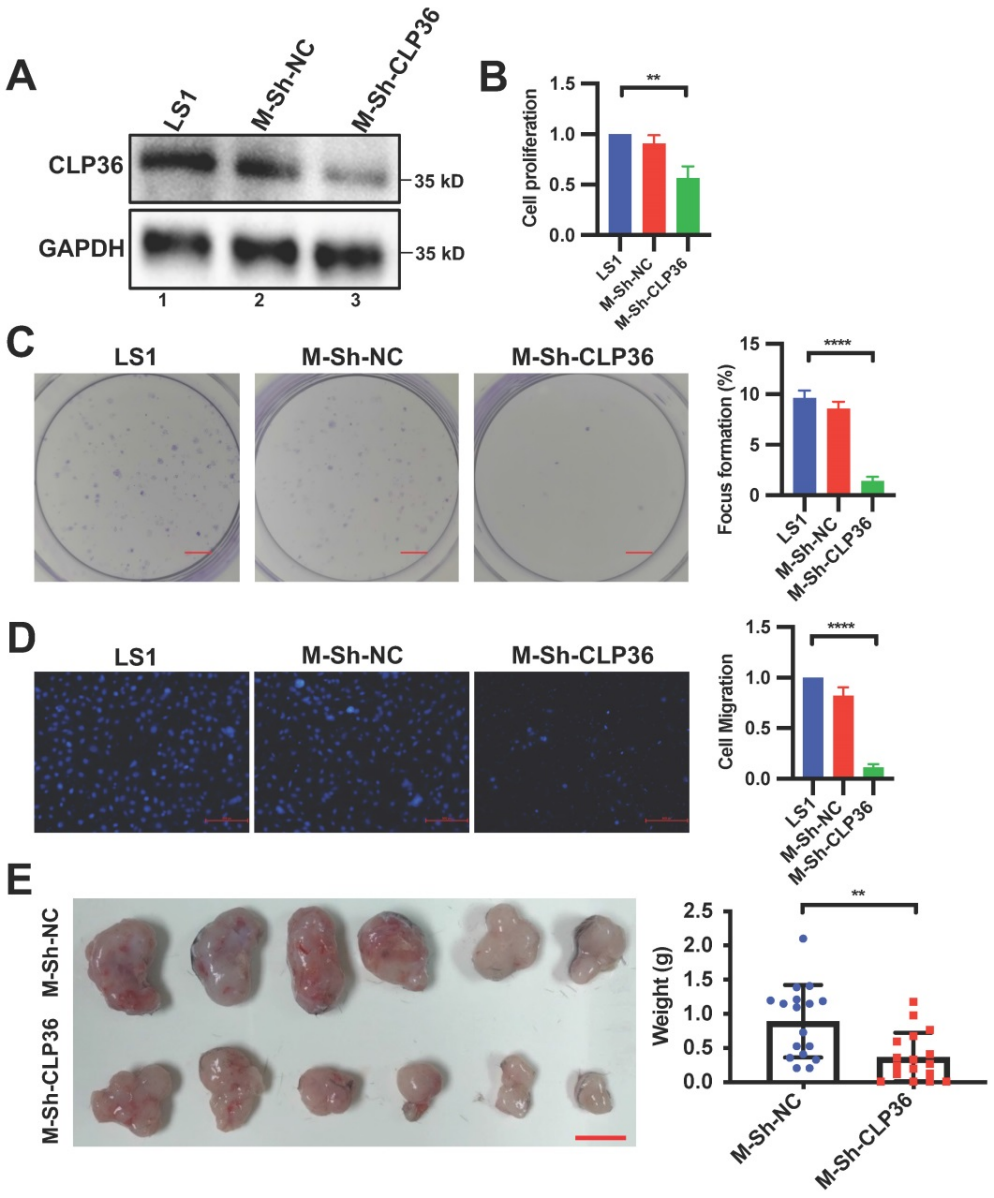
** Depletion of CLP36 reduces cell proliferation and migration *in vitro* and tumor growth *in vivo.*** Primary cells (LS1) isolated from sarcoma of the CLP36^+/+^ p53^-/-^ mice were infected with mouse control shRNA lentivirus (m-Sh-NC) or mouse CLP36 shRNA lentivirus (m-Sh-CLP36) for three days. **A** The cells (as indicated) were analyzed by Western blotting with antibodies for CLP36 and GAPDH. **B** Cell proliferation was analyzed by CCK-8 assay as described in the “Methods”. The mean absorbance of the cells (as indicated) was compared to that of the LS1 cells (normalized to 1; n = 3). **C** Focus formation assay was performed as described in the “Methods” (scale bar = 5 mm). The mean percentage of focus formation of the cells (as indicated) was compared to that of the LS1 cells (right panel). **D** Cell migration was analyzed using transwell motility chambers as described in the “Methods” (scale bar = 500 pixels). Right panel, the mean number of the cells (as indicated) migrated through the membrane was compared to that of the LS1 cells (normalized to 1; n = 3). **E** Tumors derived from subcutaneously inoculated m-Sh-NC or m-Sh-CLP36 infected cells (n = 6, scale bar = 1 cm) were shown. Right panel, the mean weight of the tumors derived from the m-Sh-CLP36 infected cells was compared to that of the tumors derived from the m-Sh-NC infected cells (n = 18 tumors for each group from three independent experiments). Data in **B**, **C** and **D** are presented as mean ± S.D. Statistical significance was calculated using one-way ANOVA with Tukey-Kramer post-hoc analysis, **p < 0.01; ****p < 0.0001. Data in the right panel of **E** were presented as mean ± SEM. Statistical significance was calculated using two-tailed unpaired Student's *t*-test, **p < 0.01.

To further test the role of CLP36 in regulation of p53 deficient sarcoma cell behavior, we isolated primary cells from sarcoma tissues of the CLP36^+/+^ p53^-/-^ mouse as described in the Methods. The primary p53 KO mouse sarcoma cells were infected with lentiviral vectors encoding mouse CLP36 shRNA (m-Sh-CLP36) or control shRNA lentivirus (m-Sh-NC). As expected, CLP36 expression was significantly reduced in the m-shCLP36 sarcoma cells (Figure 4A). Consistent with what we found in Saos-2 cells, depletion of CLP36 significantly reduced primary p53 KO mouse sarcoma cell proliferation (Figure 4B), focus formation (Figure 4C) and migration (Figure 4D). Furthermore, depletion of CLP36 impaired the tumorigenicity of the primary p53 KO sarcoma cells *in vivo* (Figure 4E). Collectively, these results suggest a crucial role of CLP36 in the promotion of malignant behavior of p53 deficient sarcoma cells *in vitro* and *in vivo*.

### CLP36 regulates YAP1 expression *in vitro* and *in vivo*

**Figure 5:**
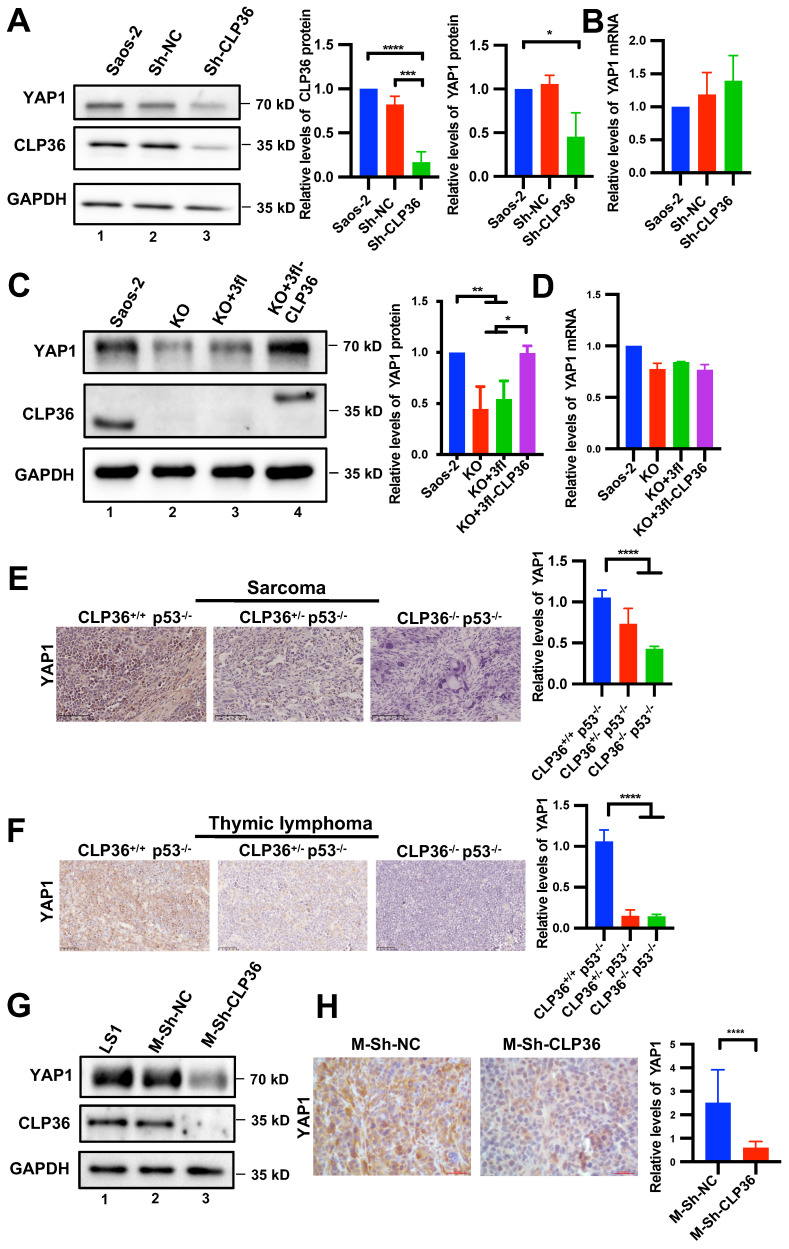
** Depletion of CLP36 reduces YAP1 expression *in vitro* and *in vivo.* A**, **B** Sh-NC or Sh-CLP36 lentivirus infected Saos-2 cells were analyzed by Western blotting for YAP1, CLP36 or GAPDH and the YAP1 level in the cells (as indicated) was compared to that in the Saos-2 cells (normalized to 1; n = 3) (**A**). The mRNA levels of YAP1 in the cells (as indicated) were analyzed by RT-PCR and compared to that in the Saos-2 cells (normalized to 1; n = 3) (**B**). **C**, **D** 3fl-CLP36 or 3fl lentivirus infected CLP36 KO Saos-2 cells were analyzed by Western blotting as indicated and the YAP1 level in the cells (as indicated) was compared to that in the Saos-2 cells (normalized to 1; n = 3) (**C**). The mRNA level of YAP1 in the cells (as indicated) was analyzed by RT-PCR and compared to that in the Saos-2 cells (normalized to 1; n = 3) (**D**). **E** Sarcoma tissues from CLP36^+/+^ p53^-/-^, CLP36^+/-^ p53^-/-^, and CLP36^-/-^ p53^-/-^ mice were stained for YAP1 (scale bar = 100 µm). The mean intensity of YAP1 of the samples (as indicated) was compared to that of the CLP36^+/+^ p53^-/-^ (n = 10 fields from two samples in the CLP36^+/+^ p53^-/-^ or CLP36^+/-^ p53^-/-^ group; n = 5 fields from one sample in the CLP36^-/-^ p53^-/-^group). **F** Thymic lymphoma tissues from CLP36^+/+^ p53^-/-^, CLP36^+/-^ p53^-/-^, and CLP36^-/-^ p53^-/-^ mice were stained for YAP1 (scale bar = 50 µm). The mean intensity of YAP1 of the samples (as indicated) was compared to that of the CLP36^+/+^ p53^-/-^ (n = 15 fields from three samples in the CLP36^+/+^ p53^-/-^ or CLP36^+/-^ p53^-/-^ group; n = 5 fields from one sample in the CLP36^-/-^ p53^-/-^group). **G** m-Sh-NC or m-Sh-CLP36 lentivirus infected LS1 cells were analyzed by Western blotting as indicated. **H** The tumors derived from cells (as indicated) were stained for YAP1 (scale bar = 20 µm). Right panel, the mean intensity of YAP1 in the m-Sh-CLP36 samples was compared to that in the m-Sh-NC samples (n = 15 fields from three samples). Data in **A**, **B**, **C** and **D** are presented as mean ± S.D. Data in **E**,** F**, and **H** are presented as mean ± SEM. Statistical significance was calculated using one-way ANOVA with Tukey-Kramer post-hoc analysis or two-tailed unpaired Student's t-test (**H**), *p < 0.05; **p < 0.01; ****p < 0.0001.

Next, we investigated the mechanism by which CLP36 functions in promotion of malignant behavior of p53 deficient sarcoma cells. YAP1 is known to crosstalk with p53 signaling and promote p53 deficiency-induced tumor growth 10, 11, 14, 42. Furthermore, consistent with previous studies 9, 43, depletion of YAP1 or TAZ significantly inhibited Saos-2 cell proliferation and migration (Figure S2). To test whether CLP36 functions in promotion of malignant behavior of p53 deficient sarcoma cells through regulation of YAP1 or TAZ, we knocked down CLP36 from Saos-2 cells and analyzed the effects on YAP1 or TAZ expression. The results showed that knockdown of CLP36 substantially reduced the protein level of YAP1 (Figure 5A) but not that of TAZ (Figure S3A), whereas the mRNA levels of neither YAP1 (Figure 5B) nor TAZ (Figure S3B) were reduced. These results suggest that CLP36 selectively regulates YAP1 expression at the protein level. To further test the effect of CLP36 on YAP1 expression, we knocked out CLP36 from Saos-2 cells using the CRISPR/Cas9 gene-editing system. KO of CLP36 also resulted in a marked reduction of the protein (Figure 5C) but not mRNA (Figure 5D) level of YAP1. Re-expression of CLP36 in the CLP36 KO cells restored to a large extent YAP1 protein expression (Figure 5C, compare lane 4 with lanes 2 and 3). Additionally, overexpression of p53 in Saos-2 cells, which inhibited CLP36 expression (Figure 1I, compare lane 3 with lanes 1 and 2; Figure S4A, compare lane 4 with lanes 1 and 2; Figure S4B, compare lane 3 with lanes 1 and 2), reduced the YAP1 level (Figure S4A, compare lane 4 with lanes 1 and 2). Knockdown of CLP36 from the p53 overexpressing Saos-2 cells further reduced the YAP1 level (Figure S4A, compare lane 5 with lane 4). Depletion of CLP36 also reduced the YAP1 level in p53 positive HT1080 fibrosarcoma cells (Figure S5A, compare lane 3 with lanes 1 and 2). Collectively, these results suggest that CLP36 plays an important role in regulation of YAP1 protein expression in sarcoma cells.

To test the effect of CLP36 on YAP1 protein expression *in vivo*, we compared the levels of YAP1 in the mouse tumor tissues that express different levels of CLP36. Immunohistochemical staining showed that while sarcoma (Figure 5E) and thymic lymphoma (Figure 5F) tissues from CLP36^+/+^ p53^-/-^ mice expressed abundant YAP1, its expression in the same tissues from CLP36^+/-^ p53^-/-^ mice, and to a greater extent that in the same tissues from CLP36^-/-^ p53^-/-^ mice, were reduced. Similarly, the YAP1 level in the tumor tissues derived from the CLP36 knockdown primary p53 KO sarcoma cells, which expressed a much lower level of YAP1 than those expressing a normal level of CLP36 (Figure 5G, compare lane 3 with lanes 1 and 2), was also significantly reduced compared with those derived from the control p53 KO sarcoma cells (Figure 5H). Collectively, these results suggest that CLP36 plays an important role in regulation of YAP1 expression in p53 deficient cells in culture as well as *in vivo*.

### CLP36-mediated regulation of YAP1 expression is dependent on AIP-4

**Figure 6:**
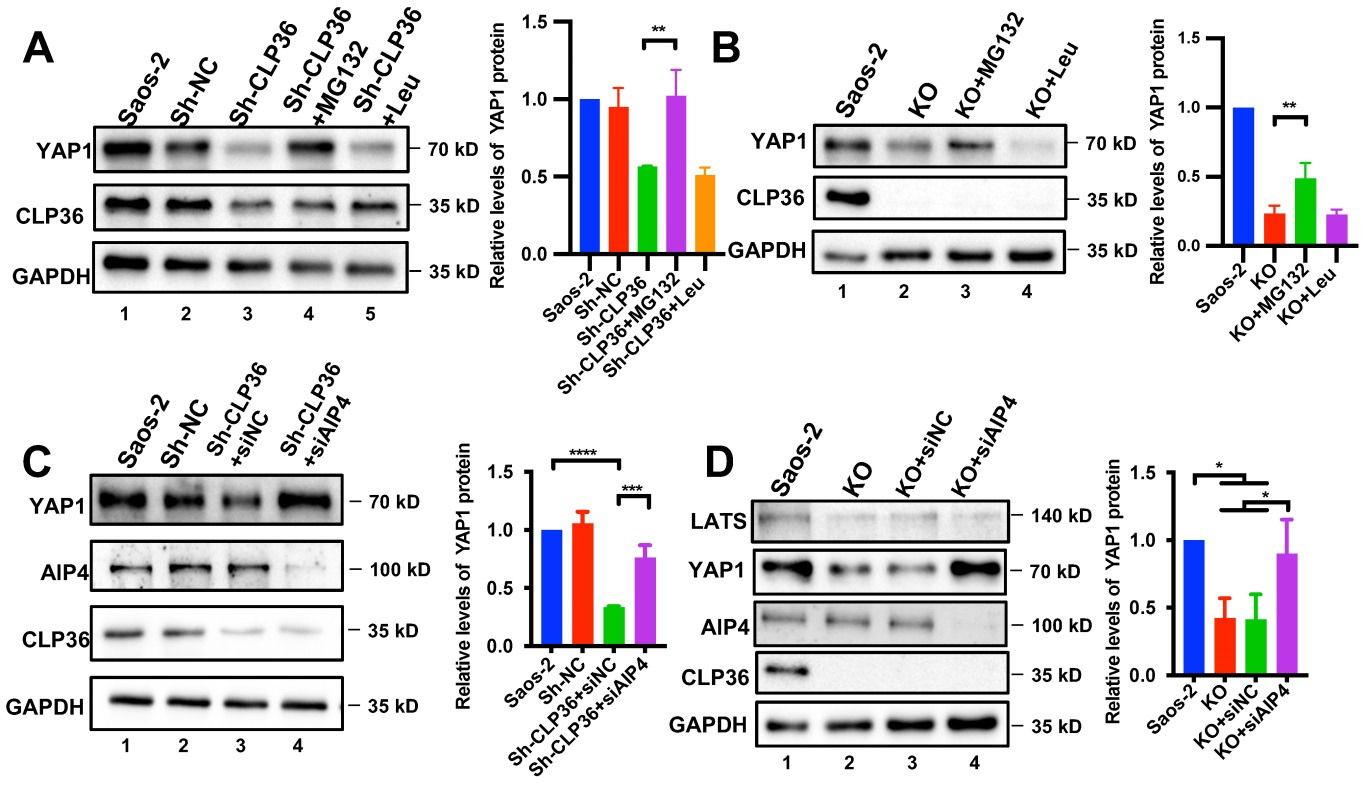
** Inhibition of proteasome or depletion of AIP-4 reverses CLP36 deficiency-induced down-regulation of YAP1 expression. A** Saos-2 cells were infected with Sh-NC or Sh-CLP36 lentivirus for three days and the Sh-CLP36 infected cells were then treated with MG132 (10 µM) or Leupeptin (10 µM) for 8h. The cells (as indicated) were analyzed by Western blotting for YAP1, CLP36 or GAPDH. Right panel, the YAP1 level in the cells (as indicated) was compared to that in the Saos-2 cells (normalized to 1; n = 3). **B** CLP36 KO Saos-2 cells were treated with MG132 (10 µM) or Leupeptin (10 µM) for 8 h. The cells (as indicated) were analyzed by Western blotting with antibodies for YAP1, CLP36 or GAPDH. Right panel, the YAP1 level in the cells (as indicated) was compared to that in the Saos-2 cells (normalized to 1; n = 3). **C** Saos-2 cells were infected with Sh-NC or Sh-CLP36 lentivirus for three days and the Sh-CLP36 infected cells were then transfected with control siRNA (siNC) or AIP-4 targeting siRNA (siAIP4) for three days. The cells (as indicated) were analyzed by Western blotting with antibodies for AIP-4, YAP1, CLP36 or GAPDH. Right panel, the YAP1 level in the cells (as indicated) was compared to that in the Saos-2 cells (normalized to 1; n = 3). **D** CLP36 KO Saos-2 cells were transfected with control siRNA (siNC) or AIP-4 targeting siRNA (siAIP4) for three days. The cells (as indicated) were analyzed by Western blotting with antibodies for AIP-4, YAP1, CLP36 or GAPDH. Right panel, the YAP1 level in the cells (as indicated) was compared to that in the Saos-2 cells (normalized to 1; n = 3). Data in **A**,** B**, **C** and **D** are presented as mean ± S.D. Statistical significance was calculated using one-way ANOVA with Tukey-Kramer post-hoc analysis, *p < 0.05; **p < 0.01; ***p < 0.001; ****p < 0.0001.

We next investigated the mechanism by which CLP36 regulates YAP1 expression. The finding that CLP36 regulates the protein but not the mRNA level of YAP1 (Figure 5) raised an intriguing possibility that CLP36 may influence YAP1 protein level through regulation of YAP1 degradation. To test this, we knocked down CLP36 from Saos-2 cells by RNAi, treated the cells with or without proteasome inhibitor MG132 or lysosome inhibitor Leupeptin, and analyzed the effects on YAP1 expression. The results showed that treatment with MG132 (Figure 6A, lane 4) but not Leupeptin (Figure 6A, lane 5) reversed the CLP36 deficiency-induced down-regulation of YAP1 expression. Similar results were obtained with CLP36 KO Saos-2 cells (Figure 6B). These results suggest that CLP36 deficiency reduces the YAP1 protein level by promoting proteasomal degradation of YAP1.

Proteasome-mediated YAP1 degradation can be regulated by Hippo/LATS-dependent or -independent signaling pathways 17, 24, 26. We first analyzed the effect of CLP36 deficiency on YAP1 phosphorylation at Ser127, a key YAP1 phosphorylation site catalyzed by LATS1. As expected, KO of CLP36 significantly reduced the YAP1 level (Figure S6A, compare lane 2 with lane 1). However, KO of CLP36 only marginally increased the ratio of Ser127 phosphorylated YAP1/total YAP1, which did not reach statistical significance (Figure S6B). Consistent with this, KO of CLP36 did not cause obvious changes of YAP1 nuclear localization (Figure S6D). The ratio of Thr1079 phosphorylated LATS1/total LATS1 was also not significantly changed in response to depletion of CLP36 (Figure S6C). These results suggest that the Hippo/LATS1 pathway probably is not the primary mediator of CLP36 deficiency-induced down-regulation of YAP1 expression. Previous studies by us and others have shown that proteasome-mediated YAP1 degradation can be regulated by F-actin- and AIP-4-dependent pathway 16, 18, 24, 26. To test whether AIP-4 is involved in this process, we depleted AIP-4 from CLP36 knockdown cells (Figure 6C, lane 4) and analyzed the effect on YAP1 expression. The results showed that depletion of AIP-4 reversed to a great extent CLP36 deficiency-induced down-regulation of YAP1 expression (Figures 6C-D, compare lane 4 with lanes 2 and 3). Similar results were obtained with CLP36 KO cells (Figure 6D and S7). In contrast to the marked increase of YAP1 level, the LATS1 level was not significantly changed in response to knockdown of AIP4 (Figure 6D, compare lane 4 with lanes 2 and 3). Depletion of β-TRCP, an E3-ligase that catalyzes ubiquitination and degradation of YAP1 in response to Hippo/LATS-dependent signaling 17, failed to block CLP36 deficiency-induced down-regulation of YAP1 (Figure S7, compare lane 4 with lane 2). Collectively, these results suggest that E3 ligase AIP-4, but not β-TRCP, is critically involved in CLP36 deficiency-induced proteasomal degradation of YAP1.

### The α-actinin-binding site is required for CLP36-mediated regulation of YAP1 expression

**Figure 7:**
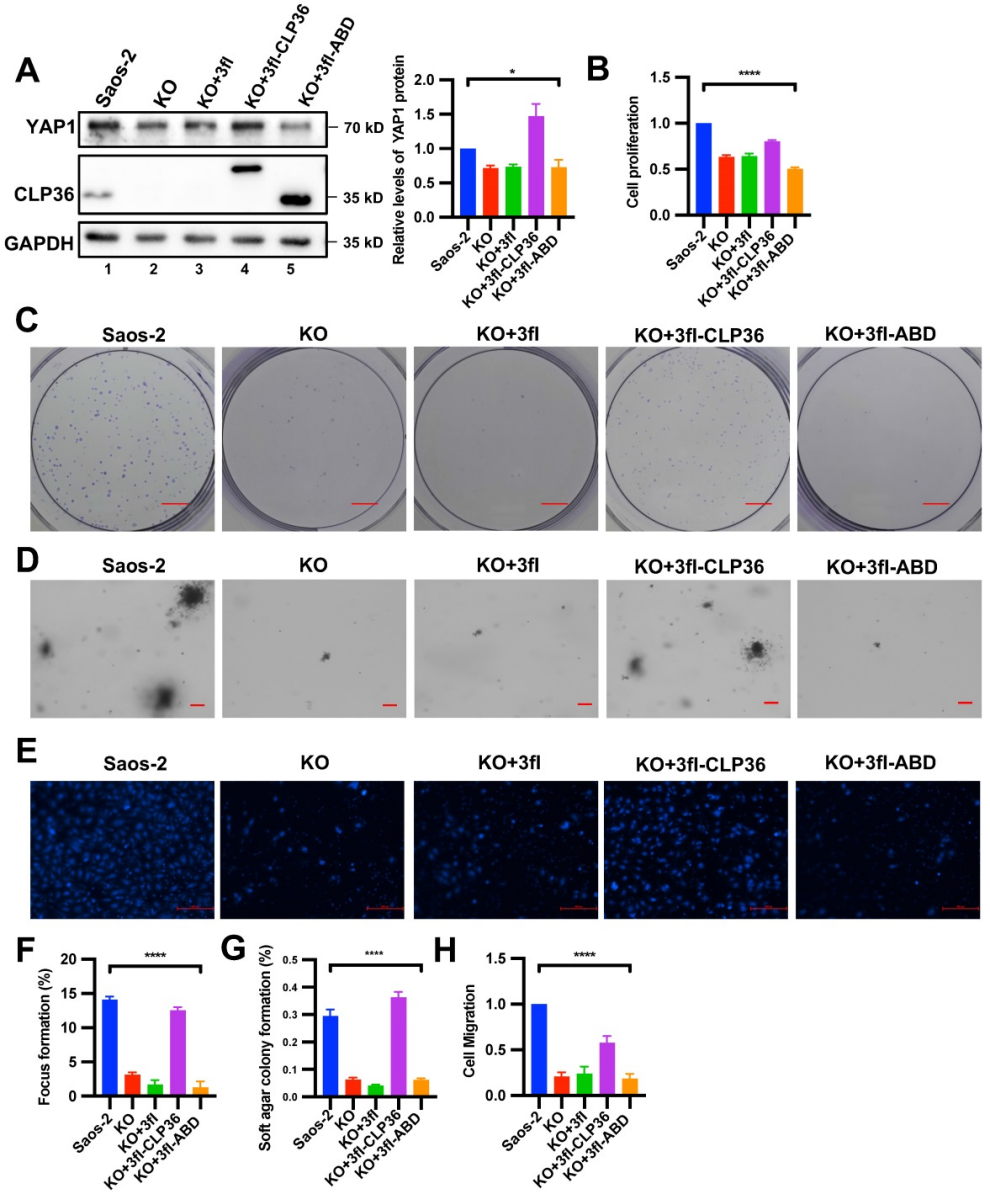
** The α-actinin-binding site is required for CLP36-mediated regulation of YAP1 expression.** CLP36 KO Saos-2 cells were infected with 3fl, 3fl-CLP36 or 3xflag-tagged α-actinin-binding defective CLP36 mutant (3fl-ABD) lentivirus for three days. **A** The cells (as indicated) were analyzed by Western blotting with antibodies for YAP1, CLP36 or GAPDH. Right panel, the YAP1 level in the cells (as indicated) was compared to that in the Saos-2 cells (normalized to 1; n = 3). **B** Cell proliferation was analyzed by CCK-8 assay as described in the “Methods”. The mean absorbance in the cells (as indicated) was compared to that in the Saos-2 cells (normalized to 1; n = 3). **C** Focus formation assay was performed as described in the “Methods” (scale bar = 5 mm). The mean percentage of focus formation in the cells (as indicated) was compared to that in the Saos-2 cells (**F**). **D** Anchorage-independent growth was analyzed by soft agar assay as described in the “Methods” (scale bar = 100 pixels). The mean percentage of colony formation in the cells (as indicated) was compared to that in the Saos-2 cells (**G**). **E** Cell migration was analyzed using transwell motility chambers as described in the “Methods” (scale bar = 500 pixels). **H** The mean number of the cells (as indicated) migrated through the membrane was compared to that of the Saos-2 cells (normalized to 1; n = 3). Data in **A**,** B**, **F**, **G** and **H** are presented as mean ± S.D. Statistical significance was calculated using one-way ANOVA with Tukey-Kramer post-hoc analysis, *p < 0.05; ****p < 0.0001.

To test whether α-actinin-binding is involved in the CLP36-mediated regulation of YAP1 expression, we expressed a 3xflag-tagged α-actinin-binding defective CLP36 mutant (3fl-ABD) 31 or 3xflag-tagged wild type CLP36 (3fl-CLP36) as a positive control in CLP36 KO p53 deficient sarcoma cells. As expected, expression of 3fl-CLP36 in CLP36 KO cells completely restored YAP1 expression (Figure 7A, lane 4). By marked contrast, expression of 3fl-ABD failed to restore YAP1 expression (Figure 7A, lane 5), suggesting that α-actinin-binding is crucial for CLP36-mediated regulation of YAP1 expression. Concomitant to the restoration of YAP1 expression, expression of 3fl-CLP36 but not the α-actinin-binding defective 3fl-ABD in CLP36 KO cells significantly reversed the CLP36 deficiency-induced inhibition on cell proliferation (Figure 7B), focus formation (Figures 7C and 7F), anchorage-independent growth (Figures 7D and 7G) and migration (Figures 7E and 7H). These results suggest that α-actinin-binding is crucial for CLP36-mediated regulation of YAP1 expression and related cellular processes.

### Down-regulation of YAP1 expression is responsible for CLP36 deficiency-induced inhibition of p53 deficient sarcoma cell proliferation, focus formation and anchorage-independent growth

**Figure 8:**
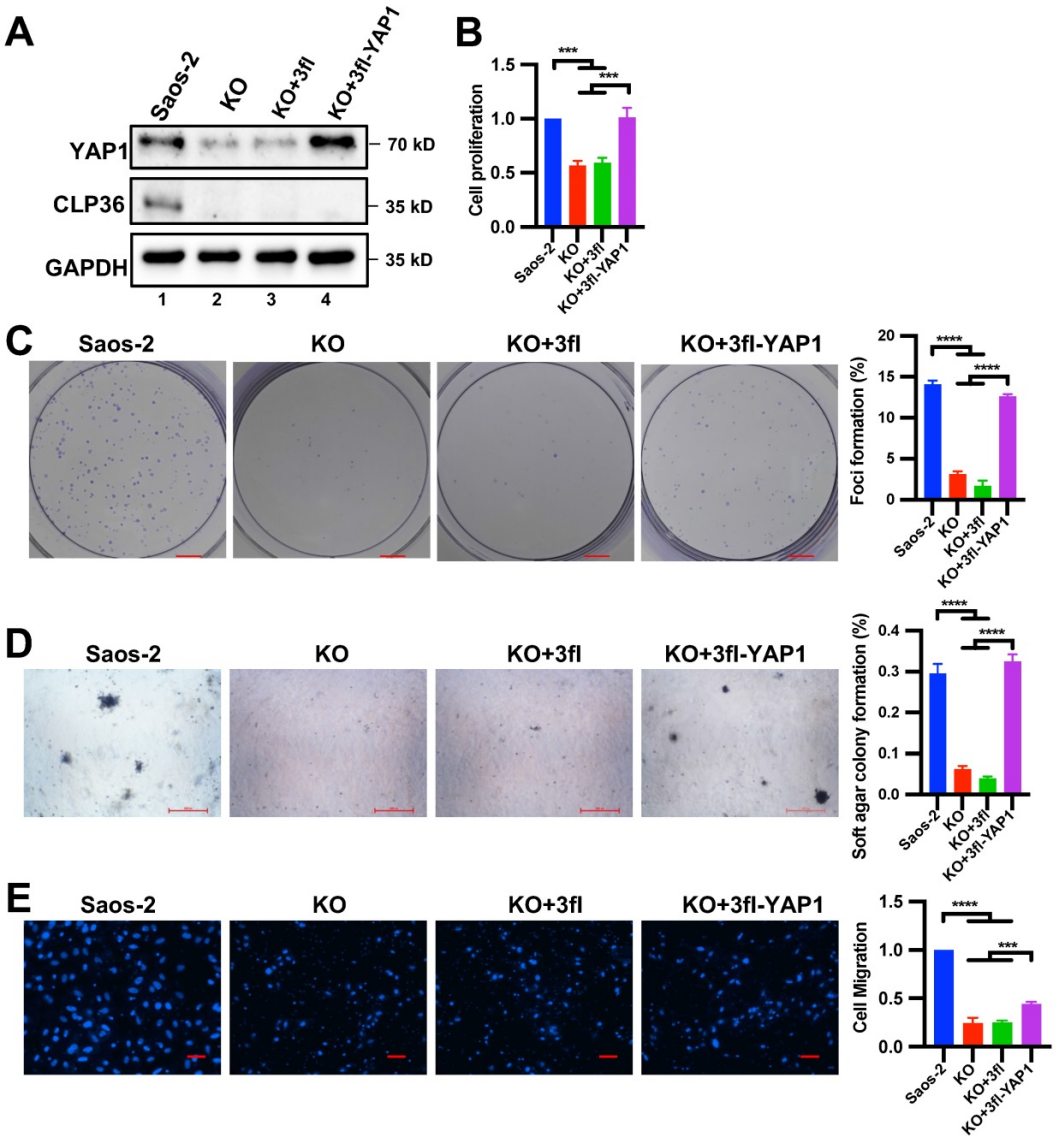
** Overexpression of YAP1 in CLP36 KO Saos-2 cells restores cell proliferation, focus formation and anchorage-independent growth.** CLP36 KO Saos-2 cells were infected with lentiviral vectors encoding 3xflag-tagged YAP1 (3fl-YAP1) or 3xflag vector (3fl) for three days. **A** The cells (as indicated) were analyzed by Western blotting with antibodies for YAP1, CLP36 or GAPDH. **B** Cell proliferation was analyzed by CCK-8 assay as described in the “Methods”. The mean absorbance of the cells (as indicated) was compared to that of the Saos-2 cells (normalized to 1; n = 3). **C** Focus formation assay was performed as described in the “Methods” (scale bar = 5 mm). The mean percentage of focus formation of the cells (as indicated) was compared to that of the Saos-2 cells (right panel). **D** Anchorage-independent growth was analyzed by soft agar assay as described in the “Methods” (scale bar = 500 pixels). The mean percentage of colony formation of the cells (as indicated) was compared to that of the Saos-2 cells (right panel). **E** Cell migration was analyzed using transwell motility chambers as described in the “Methods” (scale bar = 200 pixels). Right panel, the mean number of the cells (as indicated) migrated through the membrane was compared to that of the Saos-2 cells (normalized to 1; n = 3). Data in **B**, **C**, **D**, and **E** are presented as mean ± S.D. Statistical significance was calculated using one-way ANOVA with Tukey-Kramer post-hoc analysis, ***p < 0.001; ****p < 0.0001.

We next tested whether the down-regulation of YAP1 expression is responsible for CLP36 deficiency-induced inhibition of p53 deficient sarcoma cell proliferation, focus formation, anchorage-independent growth and migration. To do this, we increased YAP1 expression in CLP36 KO p53 deficient sarcoma cells by infecting them with lentiviral vectors encoding 3xflag-tagged YAP1 (3fl-YAP1) (Figure 8A, lane 4) or 3xflag (3fl) (Figure 8A, lane 3) as a control and tested the effects on cell behavior. The results showed that forced increase of YAP1 expression in CLP36 KO p53 deficient sarcoma cells completely restored cell proliferation (Figure 8B), focus formation (Figure 8C), and anchorage-independent growth (Figure 8D), indicating that the down-regulation of YAP1 expression is responsible for CLP36 deficiency-induced effects on these cellular processes. Of note, CLP36 deficiency-induced inhibition of p53 deficient sarcoma cell migration was only partially reversed (Figure 8E), suggesting that the defect in cell migration is caused by not only down-regulation of YAP1 expression but also alterations of other processes that are pertinent to cell migration.

## Discussion

The studies presented in this paper reveal an important role of CLP36 in p53 deficiency-induced genesis and progression of sarcoma. Several lines of evidence suggest that CLP36 acts as a relatively early and critical effector downstream of p53 during the initiation and growth of sarcoma. Firstly, increased CLP36 expression was detected in non-tumor soft tissues (e.g., muscle) of p53 deficient mice (Figure 1C), suggesting that p53 deficiency-induced increase of CLP36 expression occurs relatively early (i.e., prior to the development of histologically detectable sarcoma). Secondly, knockdown or KO of CLP36 from p53 deficient sarcoma cells was sufficient to inhibit sarcoma cell proliferation, focus formation, and anchorage-independent growth (Figures 3, 4 and S1), suggesting that the increase of CLP36 expression is critical for p53 deficiency-induced sarcoma growth. Last and perhaps most importantly, KO of CLP36 from p53 deficient mice markedly inhibited the initiation and growth of sarcoma *in vivo* (Figure 2), resulting in significant improvement of the survival of these mice.

The finding that CLP36 acts as a downstream effector of p53 raises an interesting question, namely how p53 functions in this process. The studies presented in this paper suggest that p53 can regulate CLP36 expression through multiple mechanisms. KO of p53 in mouse or depletion of p53 from p53-expressing cells in culture resulted in marked increases of both the mRNA and protein levels of CLP36 (Figures 1C-H), suggesting that p53 regulates CLP36 expression through, at least in part, a transcriptional regulation mechanism. However, despite an increase of the CLP36 mRNA level, overexpression of p53 in p53 null Saos-2 cells resulted in a significant reduction of the CLP36 protein level (Figure 1I), suggesting that at least in some cells p53 can also suppress CLP36 protein expression through a post-transcriptional regulation mechanism. Our preliminary studies showed that treatment of p53 overexpressing Saos-2 cells with MG132 or Leupeptin failed to reverse the p53 overexpression-induced reduction of the CLP36 protein level (Figure S4B), suggesting that proteasome or Leupeptin-sensitive proteases are probably not involved in this process. Clearly, future studies are required to elucidate the molecular mechanisms by which p53 regulates CLP36 expression.

What is the downstream effector of CLP36 signaling in p53 deficiency-induced initiation and growth of sarcoma? The findings presented in this paper suggest that CLP36 functions in this process primarily through promotion of YAP1 expression. Depending on the cellular context, YAP1 can either drive or suppress tumorigenesis 10. It has been well-established that, for example, p53 deficiency potentiates YAP1-mediated tumor progression 10. YAP1 overexpression and activation are closely correlated with poor tumor prognosis, especially in p53 deficient cancers 9,10. Using both *in vitro* and *in vivo* experimental approaches, we have demonstrated that CLP36 acts as a positive regulator of YAP1 expression (Figures 5, S5 and S6). Based on these studies, we proposed a model in which a signaling pathway consisting of p53, CLP36, and YAP1 is crucial for regulation of the genesis and progression of sarcoma. This signaling is activated by loss of p53, which up-regulates CLP36 expression and consequently promotes YAP1 expression, resulting in malignant transformation. This model predicts that the function of CLP36 in tumorigenesis is dependent on the function of YAP1 and provides an explanation as to why CLP36 promotes p53 deficient sarcoma growth, as p53 deficiency is known to potentiate YAP1-mediated tumor progression 10.

Our model is also consistent with previous studies showing that deletion of FoxM1, a target gene of YAP1 transcriptional coactivator, inhibited the growth of p53 null thymic lymphoma and sarcoma cells 44. Clearly, future studies are required to further test our model. It is worth noting that, in contrast to an inhibitory role of CLP36 deficiency on the proliferation of p53 deficient sarcoma cells (Figures 3B, 4B and S1B), KO of CLP36 from p53 positive HT1080 fibrosarcoma cells reduced YAP1 expression (Figure S5B) but failed to inhibit cell proliferation (Figure S5B, right panel). Similarly, we previously showed that depletion of CLP36 from p53 positive MDA-MB-231 breast cancer cells also failed to inhibit cell proliferation 31. By contrast, depletion of CLP36 from chronic myeloid leukemia cancer cells that are deficient in p53 or bear loss-of-function p53 mutations inhibited cell proliferation 45. These studies raise an interesting possibility that the function of CLP36 in promoting cancer cell proliferation, like that of YAP1, is context (i.e., p53 status)-dependent. While the current study focuses on the role of the CLP36/YAP1 signaling axis in the genesis and progression of p53 deficient sarcoma, this signaling axis may also play a role in promoting other types of cancers. In this regard, it is worth noting that both CLP36 and YAP1 have been found to promote the progression of glioma, breast cancer, and CML 31, 45-48. It will be interesting to test whether the CLP36/YAP1 signaling axis delineated in the current study also operates in other types of cancers and how it cooperates with p53 signaling in regulation of the progression of these types of cancer.

YAP1 regulates not only cell proliferation but also cell migration. Indeed, overexpression of YAP1 in CLP36- and p53-deficient sarcoma cells partially reversed the CLP36 deficiency-induced down-regulation of cell migration (Figure 8E), suggesting that p53/CLP36/YAP1 signaling pathway may also contribute to the regulation of cell migration in these cells. However, unlike cell proliferation, focus formation, and anchorage-independent growth, which were restored entirely in response to overexpression of YAP1 (Figures 8B-D), the effect of YAP1 overexpression on CLP36 deficient sarcoma cell migration was somewhat limited (Figure 8E). This may reflect the fact that CLP36 regulates not only YAP1 but also other signaling pathways that are pertinent to cell migration. In this regard, it is worth noting that CLP36 has been found to regulate Cdc42 31 and p75 neurotrophin receptor 48, which are known to be crucial for cell migration.

The findings presented in this paper not only reveal a crucial role of CLP36 in regulation of YAP1 expression but also shed light on the molecular mechanism by which CLP36 functions in this process. Several lines of evidence suggest that CLP36 regulates YAP1 expression through control of E3 ligase AIP-4-mediated proteasomal degradation of YAP1. Firstly, loss of CLP36 reduces the protein but not the mRNA level of YAP1 (Figures 5A-D). Secondly, inhibition of proteasome but not that of lysosome reversed CLP36 deficiency-induced down-regulation of YAP1 expression (Figures 6A-B). Thirdly, it has been shown that CLP36 binds to α-actinin and localizes to F-actin, which in turn can regulate E3 ligase AIP-4-mediated proteasomal degradation of YAP1 24, 26. The results from the rescue experiments with α-actinin-binding defective CLP36 mutant indicate that α-actinin-binding is required for CLP36-mediated regulation of YAP1 expression, suggesting that CLP36 probably regulates AIP4-dependent degradation of YAP1 indirectly (e.g., through F-actin). Consistent with this, immunoprecipitation experiments failed to detect a physical association between CLP36 and YAP1 (Figure S8). Finally, depletion of AIP-4 completely blocked CLP36 deficiency-induced down-regulation of YAP1 expression (Figures 6C-D). In contrast to the prominent role of α-actinin-binding and AIP-4 in CLP36-mediated regulation of YAP1 expression, the Hippo/LATS signaling pathway does not seem to play a major role in this process (Figure S6). Consistent with this, depletion of β-TRCP, an E3-ligase that is known to be involved in Hippo/LATS-mediated regulation of YAP1 expression 17, failed to block CLP36 deficiency-induced down-regulation of YAP1 expression in p53 deficient sarcoma cells (Figure S7). It is worth noting, however, the finding that the Hippo/LATS signaling pathway is not directly involved in the p53/CLP36-mediated regulation of YAP1 expression does not necessarily exclude the possibility that Hippo/LATS signaling plays a role in the development and progression of sarcoma. Indeed, abundant YAP1 was detected in not only p53 negative but also p53 positive sarcomas (Figure S9), suggesting that other YAP1 regulators (e.g., components of the Hippo/LATS signaling pathway) may also contribute to the up-regulation of YAP1 expression and signaling in at least a subset of this disease.

In summary, we have demonstrated that CLP36 acts as a critical mediator linking p53 deficiency to sarcoma progression and shed light on the molecular mechanism through which CLP36 functions in this process. Therapeutic targeting the CLP36/YAP1 signaling axis may provide an effective strategy for alleviation of p53 deficient sarcoma progression.

## Supplementary Material

Supplementary figures and table.

## Abbreviations

AIP4: atrophin-1-interacting protein 4
β-TRCP: β-Transducin repeat-containing proteins
CLP36: carboxyl terminal LIM domain protein of 36 kDa
CML: chronic myeloid leukemia
FOXM1: forkhead box m1
IHC: immunohistochemistry
KO: knockout
LATS1: large tumor suppressor kinase 1
RT-qPCR: quantitative real-time PCR
YAP1: yes-associated protein 1
