# Supplementary material for: CLP36 promotes p53 deficient sarcoma progression through suppression of atrophin-1 interacting protein-4 (AIP-4)-dependent degradation of YAP1
Source: Theranostics. 2022 Jul 4;12(11):5051–68. doi: 10.7150/thno.72365 (PMC9274740; doi:10.7150/thno.72365)
Supplement: Supplementary file 1 — Supplementary figures and table. [file thnov12p5051s1.pdf]

Supplementary Material

Supplementary figures

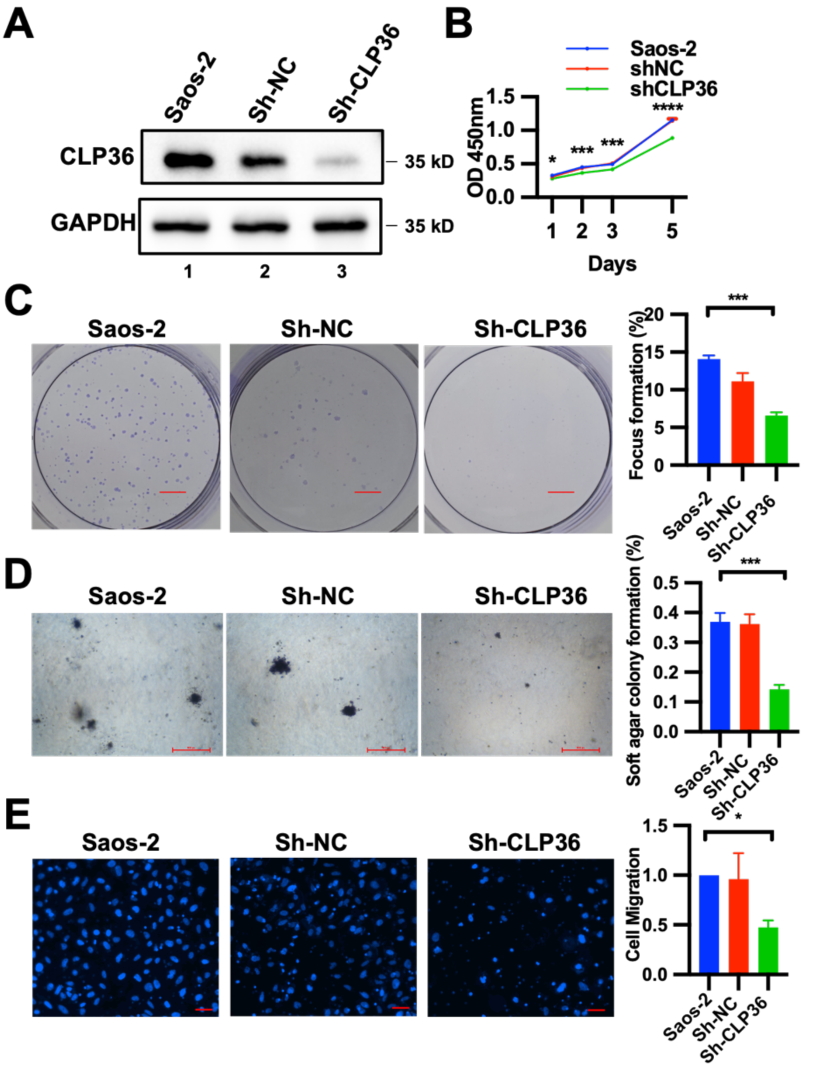

Figure S1. Knockdown of CLP36 reduces p53 deficient sarcoma cell proliferation and migration

Saos-2 cells were infected with Sh-NC or Sh-CLP36 lentivirus for five days. **A** The cells (as indicated) were analyzed by Western blotting with antibodies for CLP36 or GAPDH. **B** Cell proliferation was analyzed by CCK-8 assay as described in the

"Methods". The absorbance of cells at 450nm on the 1st, 2nd, 3rd and 5th day was quantified. The mean absorbance of the Sh-CLP36 infected cells was compared to that of the Saos-2 cells. **C** Focus formation assay was performed as described in the "Methods" (scale bar = 5 mm). The mean percentage of focus formation of the cells (as indicated) was compared to that of the Saos-2 cells (right panel). **D** Anchorage-independent growth was analyzed by soft agar assay as described in the "Methods" (scale bar = 500 pixels). The mean percentage of colony formation of the cells (as indicated) was compared to that of the Saos-2 cells (right panel). **E** Cell migration was analyzed using transwell motility chambers as described in the "Methods" (scale bar = 200 pixels). Right panel, the mean number of the cells (as indicated) migrated through the membrane was compared to that of the Saos-2 cells (normalized to 1; n = 3). Data in **B**, **C**, **D**, and **E** are presented as mean ± S.D. Statistical significance was calculated using one-way ANOVA with Tukey–Kramer post-hoc analysis, *$p < 0.05$; ***$p < 0.001$; ****$p < 0.0001$.

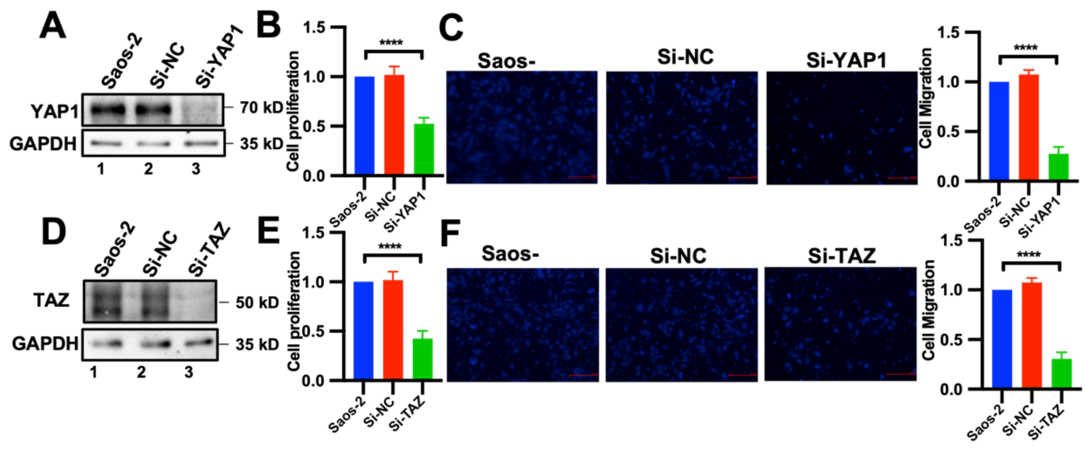

Figure S2. Depletion of either YAP1 or TAZ inhibits cell proliferation and migration of Saos-2 cells.

A, **B**, **C** Saos-2 cells were transfected with Si-NC or Si-YAP1 for three days. The cells (as indicated) were analyzed by Western blotting with antibodies for YAP1 or GAPDH (**A**). Cell proliferation was analyzed by CCK-8 assay as described in the "Methods". The mean absorbance of the Si-YAP1 cells was compared to that of the Saos-2 cells (**B**). Cell migration was analyzed using transwell motility chambers as described in the "Methods" (scale bar = 200 pixels). Right panel, the mean number of the cells (as indicated) migrated through the membrane was compared to that of the Saos-2 cells (normalized to 1; n = 3) (**C**). **D**, **E**, **F** Saos-2 cells were transfected with Si-NC or Si-TAZ for three days. The cells (as indicated) were analyzed by Western blotting with antibodies for TAZ or GAPDH (**D**). Cell proliferation was analyzed by CCK-8 assay as described in the "Methods". The mean absorbance of the Si-TAZ cells was compared to that of the Saos-2 cells (**E**). Cell migration was analyzed using transwell motility chambers as described in the "Methods" (scale bar = 200 pixels). Right panel, the mean number of the cells (as indicated) migrated through the membrane was compared to that of the Saos-2 cells (normalized to 1; n = 3) (**F**). Data in **B**, **C**, **E**, and **F** are presented as mean ± S.D. Statistical significance was calculated using one-way ANOVA with Tukey–Kramer post-hoc analysis, $****p < 0.0001$.

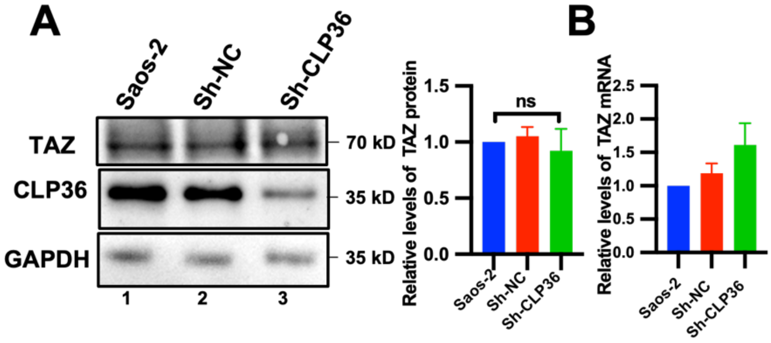

Figure S3. Depletion of CLP36 does not significantly alter TAZ expression

Sh-NC or Sh-CLP36 lentivirus infected Saos-2 cells were analyzed by Western blotting with antibodies for TAZ, CLP36 or GAPDH and the TAZ level in the cells (as indicated) was compared to that in the Saos-2 cells (normalized to 1; n = 3) (**A**). The mRNA levels of TAZ in the cells (as indicated) were analyzed by RT-PCR and compared to that in the Saos-2 cells (normalized to 1; n = 3) (**B**). Data is presented as mean ± S.D. Statistical significance was calculated using one-way ANOVA with Tukey–Kramer post-hoc analysis, ns, no significance.

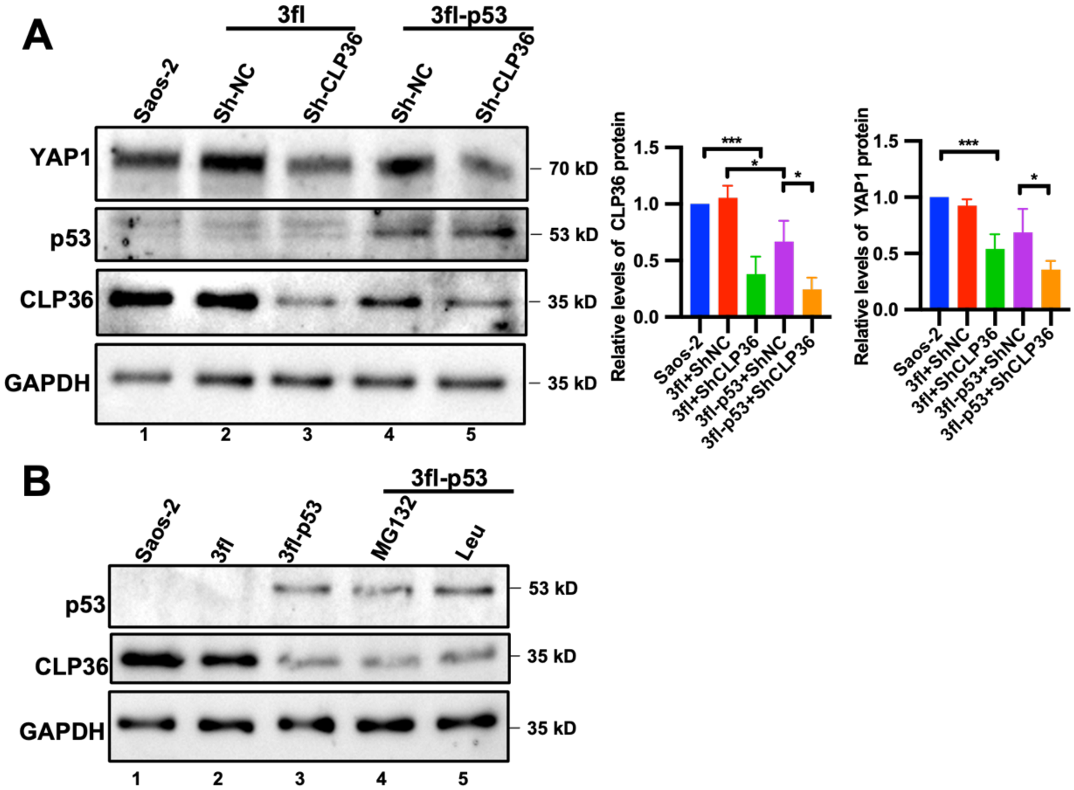

Figure S4. Overexpression of p53 in p53 deficient sarcoma cells reduces CLP36 and YAP1 expression

Saos-2 cells were infected with lentiviral vectors encoding 3xflag-tagged p53 (3fl-p53) or 3xflag vector (3fl) for three days. **A** The cells (as indicated) were then infected with Sh-NC or Sh-CLP36 lentivirus for three days. Left panel, the cells were analyzed by Western blotting with antibodies for YAP1, CLP36 or GAPDH. Middle and right panels, the levels of CLP36 and YAP1 in the cells (as indicated) were compared to those in the Saos-2 cells (normalized to 1; n = 3). **B** The cells (as indicated) were treated with MG132 (10 uM) or Leupeptin (10 uM) for 8 hours. The cells (as indicated) were analyzed by Western blotting with antibodies recognizing p53, CLP36 or GAPDH.

Data in **A** is presented as mean ± S.D. Statistical significance was calculated using one-way ANOVA with Tukey–Kramer post-hoc analysis, $*p < 0.05$; $***p < 0.001$.

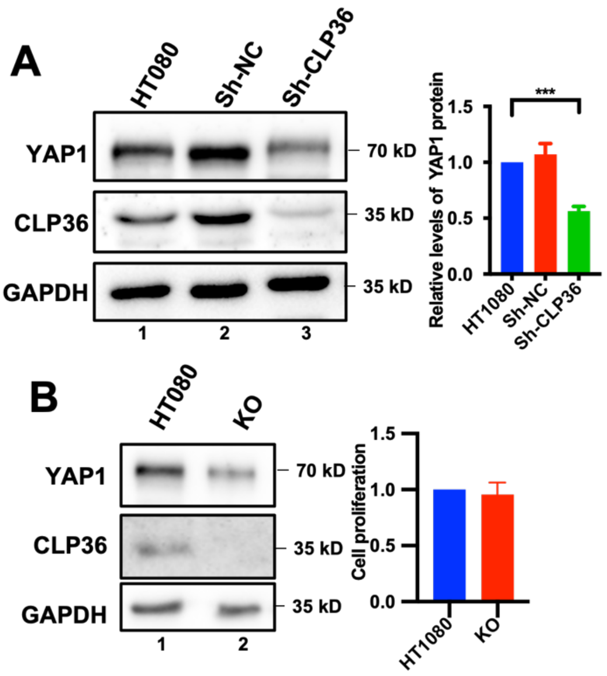

Figure S5. Depletion of CLP36 from p53 expressing HT1080 fibrosarcoma cells diminishes YAP1 expression but fails to inhibit cell proliferation

A HT1080 cells were infected with Sh-NC or Sh-CLP36 lentivirus for three days. The cells were analyzed by Western blotting with antibodies for YAP1, CLP36 or GAPDH. Right panel, the YAP1 level in the Sh-CLP36 or Sh-NC infected cells was compared to that in the HT1080 cells (normalized to 1; n = 3). **B** CLP36 KO and wild type HT1080 cells were analyzed by Western blotting with antibodies for CLP36, YAP1 or GAPDH. Right panel, CLP36 KO or HT1080 cells were seeded in 10cm dishes at the density of 1×10^5 cells/dish, cultured in the basal growth medium for three days and then the cell numbers were counted. The number of the CLP36 KO cells was

compared to that of the wild type HT1080 cells (normalized to 1; n = 3). Data in **A** is presented as mean ± S.D. Statistical significance was calculated using one-way ANOVA with Tukey–Kramer post-hoc analysis, ***$p < 0.001$.

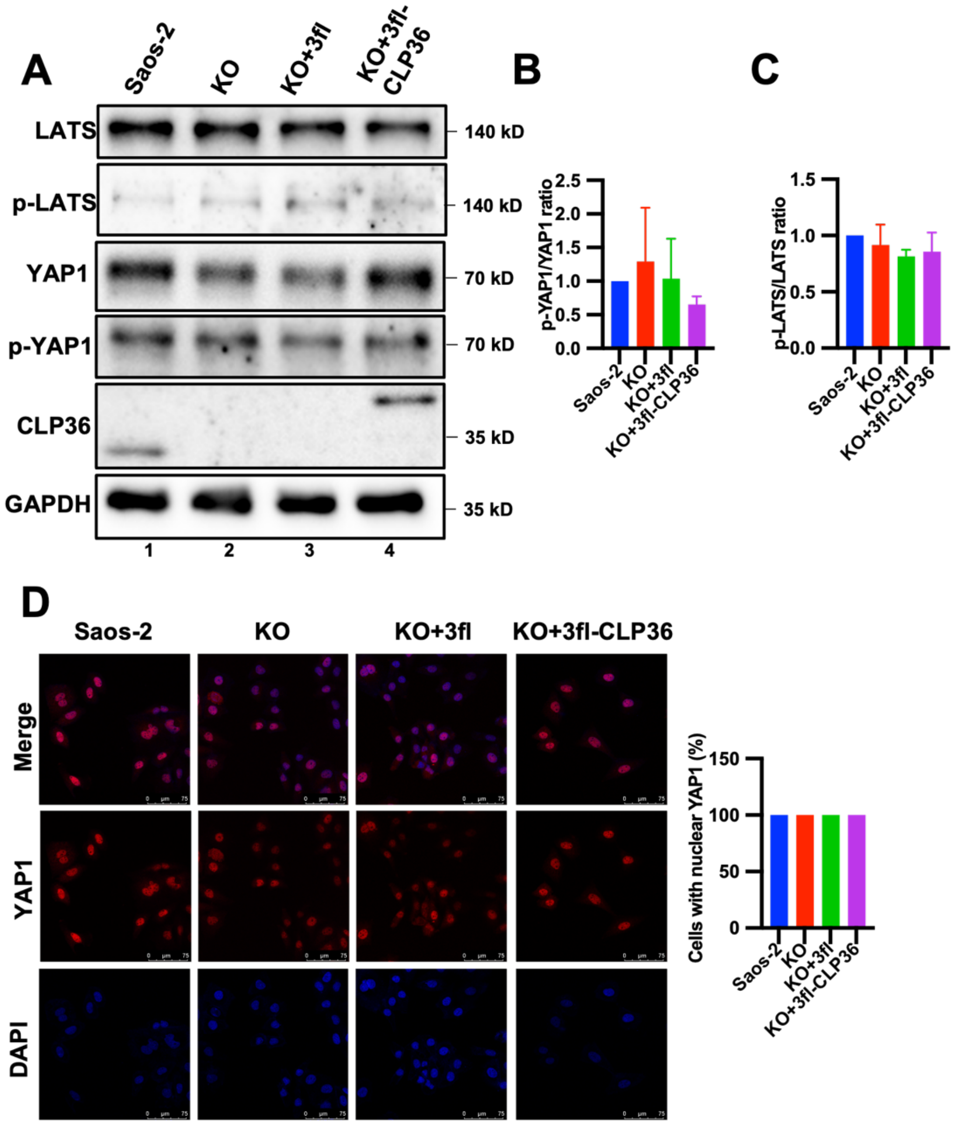

Figure S6. Depletion of CLP36 does not significantly alter LATS Thr1079 phosphorylation, YAP1 Ser127 phosphorylation and YAP1 subcellular localization

CLP36 KO Saos-2 cells were infected with 3fl-CLP36 or 3fl lentivirus for three days. **A** The cells (as indicated) were analyzed by Western blotting with antibodies for YAP1, p-YAP1 (Ser127), LATs, p-LATs (Thr1079), CLP36, or GAPDH. The ratios of Ser127-phosphorylated YAP1/YAP1 (**B**) and Thr1079-phosphorylated LATS/LATS (**C**) in the CLP36 KO Saos-2 cells were compared to those in the wild type Saos-2 cells (normalized to 1; n = 3). **D** The cells were immunofluorescent stained with DAPI (blue) and antibodies for YAP1 (red) (Scale bar = 75 μm). Right panel, the percentages of the cells (as indicated in the figure) with positive nuclear YAP1 staining were calculated as described in the "Methods".

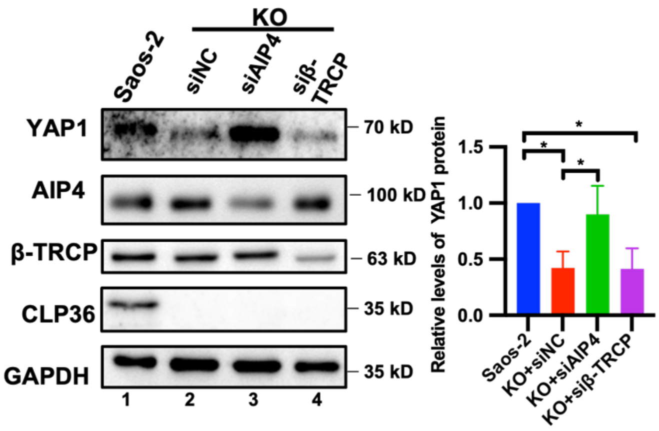

Figure S7. Depletion of AIP-4 but not that of β-TRCP reverses CLP36 deficiency-induced down-regulation of YAP1 expression

CLP36 KO Saos-2 cells were transfected with control siRNA (siNC), AIP-4 targeting siRNA (siAIP4), or siβ-TRCP targeting siRNA (siβ-TRCP) for three days. The cells (as indicated) were analyzed by Western blotting with antibodies for YAP1, AIP-

4, β-TRCP, CLP36 or GAPDH. Right panel, the YAP1 level in the cells (as indicated) was compared to that in the wild type Saos-2 cells (normalized to 1; n = 3). Data is presented as mean ± S.D. Statistical significance was calculated using one-way ANOVA with Tukey–Kramer post-hoc analysis, *$p < 0.05$.

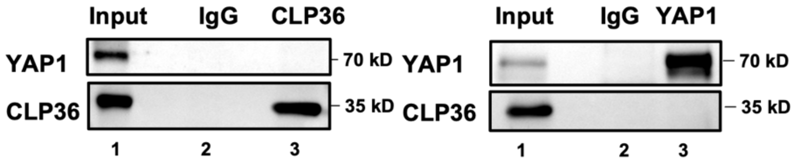

Figure S8. CLP36 is not physically associated with YAP1

The association between CLP36 and YAP1 in Saos-2 cells were analyzed by co-IP with antibodies for CLP36 (left panel) or YAP1 (right panel) as described in the "Methods". The cell lysate (lane 1), control IgG (lane 2) and anti-CLP36 (lane 3 in the left panel) or anti-YAP1 (lane 3 in the right panel) IP samples were analyzed by Western blotting with antibodies recognizing YAP1 or CLP36 as indicated.

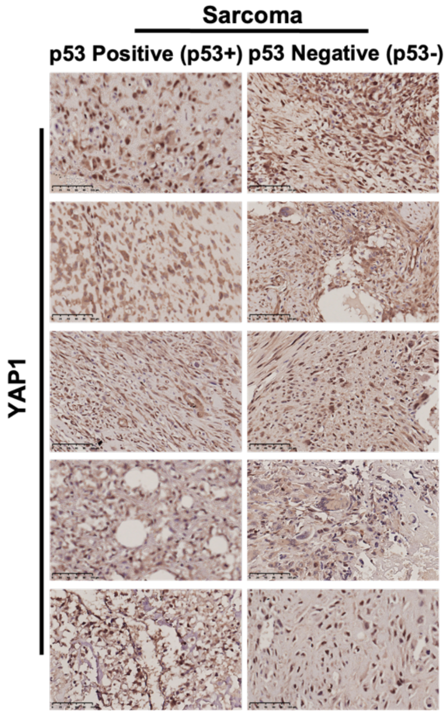

Figure S9. YAP1 is highly expressed in both p53 positive and negative osteosarcoma

Human osteosarcoma tissues from a tissue microarray (Tbsbio, Xi'an, China) were immunohistochemically stained with anti-YAP1 antibodies as described in the "Methods" (scale bar = 100 μm). Representative images of YAP1 staining from five p53 positive (tissue ID: Lbn050044B002, Lbn040053B001, Lbn080032B001, Lbn070028B001, Lbn020075B004) and five p53 negative (tissue ID: Lbn020058B002, Lbn020002B001, Lbn030133B001, Lbn050128B004, Lbn040007B001) osteosarcoma tissues are shown in the figure. The clinical information of the tissue samples was shown in Supplementary Table 1.

Supplementary Table 1. Information of the tissue array panel

No.	Age	Sex	Organ	Pathology diagnosis	TNM	Stage	Tissue ID.
1	12	F	Bone	Osteosarcoma of femur	T2N0M0 G3	IIB	Lbn070027B001
2	12	F	Bone	Osteosarcoma of femur	T2N0M0 G3	IIB	Lbn070027B001
3	14	F	Bone	Osteosarcoma of left tibia superior segment	T2N0M0 G4	IIIB	Lbn050122B001
4	14	F	Bone	Osteosarcoma of left tibia superior segment	T2N0M0 G4	IIIB	Lbn050122B001
5	17	M	Bone	Osteosarcoma of left femur inferior segment	T2N0M0 G3	IIB	Lbn050022B003
6	17	M	Bone	Osteosarcoma of left femur inferior segment	T2N0M0 G3	IIB	Lbn050022B003
7	28	M	Bone	Osteosarcoma of left femur	T2N0M0 G1	IB	Lbn020010B004
8	28	M	Bone	Osteosarcoma of left femur	T2N0M0 G1	IB	Lbn020010B004

9	12	M	Bone	Osteosarcoma of left femur inferior segment	T2N0M0 G3	IIB	Lbn020058B002
10	12	M	Bone	Osteosarcoma of left femur inferior segment	T2N0M0 G3	IIB	Lbn020058B002
11	32	F	Bone	Osteosarcoma of left femur inferior segment	T2N0M0 G3	IIB	Lbn050044B002
12	32	F	Bone	Osteosarcoma of left femur inferior segment	T2N0M0 G3	IIB	Lbn050044B002
13	15	M	Bone	Osteosarcoma of right femur	T2N0M0 G3	IIB	Lbn040053B001
14	15	M	Bone	Osteosarcoma of right femur	T2N0M0 G3	IIB	Lbn040053B001
15	46	M	Bone	Osteosarcoma of right femur (sparse)	T2N0M0 G2	IB	Lbn090016B003
16	46	M	Bone	Osteosarcoma of right femur	T2N0M0 G2	IB	Lbn090016B003
17	14	F	Bone	Osteosarcoma of left tibia superior segment	T2N0M0 G3	IIB	Lbn040125B002
18	14	F	Bone	Osteosarcoma of left tibia superior segment	T2N0M0 G3	IIB	Lbn040125B002
19	14	M	Bone	Osteosarcoma of left face	T2N0M0		Lbn020002B001
20	14	M	Bone	Osteosarcoma of left face	T2N0M0		Lbn020002B001
21	16	F	Bone	Osteoblastic osteosarcoma of left femur	T2N0M0 G2	IB	Lbn080029B001
22	16	F	Bone	Osteoblastic osteosarcoma of left femur	T2N0M0 G2	IB	Lbn080029B001
23	12	F	Bone	Osteosarcoma of right femur	T1N0M0 G3	IIA	Lbn040104B003
24	12	F	Bone	Osteosarcoma of right femur	T1N0M0 G3	IIA	Lbn040104B003
25	15	M	Bone	Osteosarcoma of right femur inferior segment	T2N0M0 G3	IIB	Lbn030117B001
26	15	M	Bone	Osteosarcoma of right femur inferior segment	T2N0M0 G3	IIB	Lbn030117B001
27	27	M	Bone	Fibroblastic osteosarcoma of humerus	T2N0M0 G3	IIB	Lbn030133B001

28	27	M	Bone	Fibroblastic osteosarcoma of humerus	T2N0M0 G3	IIB	Lbn030133B001
29	41	F	Bone	Osteosarcoma of right humerus	T1N0M0 G2	IA	Lbn100006B006
30	41	F	Bone	Osteosarcoma of right humerus	T1N0M0 G2	IA	Lbn100006B006
31	18	M	Bone	Osteosarcoma of right humerus	T2N0M0 G2	IB	Lbn050128B004
32	18	M	Bone	Osteosarcoma of right humerus	T2N0M0 G2	IB	Lbn050128B004
33	20	F	Bone	Osteosarcoma of right tibia superior segment	T2N0M0 G3	IIB	Lbn080032B001
34	20	F	Bone	Osteosarcoma of right tibia superior segment	T2N0M0 G3	IIB	Lbn080032B001
35	10	M	Bone	Osteosarcoma of right tibia	T1N0M0 G2	IA	Lbn020008B004
36	10	M	Bone	Osteosarcoma of right tibia	T1N0M0 G2	IA	Lbn020008B004
37	17	F	Bone	Osteosarcoma of right distal tibia (degeneration)	T2N0M0 G3	IIB	Lbn040026B002
38	17	F	Bone	Osteosarcoma of right distal tibia (degeneration)	T2N0M0 G3	IIB	Lbn040026B002
39	19	M	Bone	Osteosarcoma of left thigh	T2N0M0 G3	IIB	Lbn040007B001
40	19	M	Bone	Osteosarcoma of left thigh	T2N0M0 G3	IIB	Lbn040007B001
41	18	F	Bone	Osteosarcoma of left distal femur			Lbn030032B002
42	18	F	Bone	Osteosarcoma of left distal femur			Lbn030032B002
43	19	M	Bone	Osteosarcoma of right femur (sparse)	T2N0M0 G3	IIB	Lbn040027B002
44	19	M	Bone	Osteosarcoma of right femur	T2N0M0 G3	IIB	Lbn040027B002
45	56	F	Bone	Osteosarcoma of left frontal	T2N0M0 G3	IIB	Lbn140030B002
46	56	F	Bone	Osteosarcoma of left frontal	T2N0M0 G3	IIB	Lbn140030B002
47	51	M	Bone	Osteosarcoma of right fibula	T2N0M0 G3	IIB	Lbn040098B001
48	51	M	Bone	Osteosarcoma of right fibula	T2N0M0 G3	IIB	Lbn040098B001
49	21	M	Bone	Osteosarcoma of popliteal fossa	T1aN0M0	IA	Lbn090011B001
50	21	M	Bone	Osteosarcoma of popliteal fossa	T1aN0M0	IA	Lbn090011B001

51	33	M	Bone	Osteosarcoma of left upper jaw	T1N0M0 G2	IA	Lbn090013B002
52	33	M	Bone	Osteosarcoma of left upper jaw	T1N0M0 G2	IA	Lbn090013B002
53	15	M	Bone	Osteosarcoma of lower jaw	T2N1M0 G2	IVB	Lbn090019B004
54	15	M	Bone	Osteosarcoma of lower jaw	T2N1M0 G2	IVB	Lbn090019B004
55	55	M	Bone	Osteosarcoma of right Lower rib			Lbn090004B001
56	55	M	Bone	Osteosarcoma of right Lower rib			Lbn090004B001
57	28	F	Bone	Osteosarcoma of right femur	T2N0M0 G3	IIB	Lbn040051B002
58	28	F	Bone	Osteosarcoma of right femur	T2N0M0 G3	IIB	Lbn040051B002
59	17	M	Bone	Osteoblastic osteosarcoma of right femur superior segment	T2N0M0 G3	IIB	Lbn050109B001
60	17	M	Bone	Osteoblastic osteosarcoma of right femur superior segment	T2N0M0 G3	IIB	Lbn050109B001
61	13	F	Bone	Osteosarcoma of left femur inferior segment	T2N0M0 G2	IB	Lbn020034B004
62	13	F	Bone	Osteosarcoma of left femur inferior segment	T2N0M0 G2	IB	Lbn020034B004
63	17	M	Bone	Osteosarcoma of left tibia	T2N0M0 G2	IB	Lbn070028B001
64	17	M	Bone	Osteosarcoma of left tibia	T2N0M0 G2	IB	Lbn070028B001
65	23	F	Bone	Osteosarcoma of left femur	T2N0M0 G3	IIB	Lbn020076B004
66	23	F	Bone	Osteosarcoma of left femur	T2N0M0 G3	IIB	Lbn020076B004
67	31	F	Bone	Osteosarcoma of left femur	T2N0M0		Lbn020025B003
68	31	F	Bone	Osteosarcoma of left femur	T2N0M0		Lbn020025B003
69	14	M	Bone	Osteosarcoma of right femur inferior segment	T2N0M0 G2	IB	Lbn020066B013
70	14	M	Bone	Osteosarcoma of right femur inferior segment	T2N0M0 G2	IB	Lbn020066B013

71	16	M	Bone	Osteosarcoma of right femur	T2N0M0		Lbn020038B005
72	16	M	Bone	Osteosarcoma of right femur	T2N0M0		Lbn020038B005
73	42	M	Bone	Osteosarcoma of left femur	T2N0M0 G3	IIB	Lbn040129B004
74	42	M	Bone	Osteosarcoma of left femur	T2N0M0 G3	IIB	Lbn040129B004
75	19	M	Bone	Osteosarcoma of left calf fibula	T2N0M0		Lbn020075B004
76	19	M	Bone	Osteosarcoma of left calf fibula	T2N0M0		Lbn020075B004
77	37	F	Bone	Osteosarcoma of right femur inferior segment	T1N0M0 G2	IA	Lbn020043B005
78	37	F	Bone	Osteosarcoma of right femur inferior segment	T1N0M0 G2	IA	Lbn020043B005
79	50	F	Bone	Osteosarcoma of right femur inferior segment	T1N0M0 G3	IIA	Lbn080028B001
80	50	F	Bone	Osteosarcoma of right femur inferior segment	T1N0M0 G3	IIA	Lbn080028B001
